# Molecular Modeling of µ Opioid Receptor Ligands with Various Functional Properties: PZM21, SR-17018, Morphine, and Fentanyl—Simulated Interaction Patterns Confronted with Experimental Data

**DOI:** 10.3390/molecules25204636

**Published:** 2020-10-12

**Authors:** Sabina Podlewska, Ryszard Bugno, Lucja Kudla, Andrzej J. Bojarski, Ryszard Przewlocki

**Affiliations:** 1 Department of Technology and Biotechnology of Drugs, Jagiellonian University Medical College, 9 Medyczna Street, 30-688 Cracow, Poland; smusz@if-pan.krakow.pl; 2 Maj Institute of Pharmacology, Polish Academy of Sciences, 12 Smętna Street, 31-343 Cracow, Poland; bugno@if-pan.krakow.pl (R.B.); kudla@if-pan.krakow.pl (L.K.); bojarski@if-pan.krakow.pl (A.J.B.)

**Keywords:** µ opioid receptor, molecular dynamics, docking, interaction fingerprints, biased agonists, SR-17018, PZM21, morphine, fentanyl

## Abstract

Molecular modeling approaches are an indispensable part of the drug design process. They not only support the process of searching for new ligands of a given receptor, but they also play an important role in explaining particular activity pathways of a compound. In this study, a comprehensive molecular modeling protocol was developed to explain the observed activity profiles of selected µ opioid receptor agents: two G protein-biased µ opioid receptor agonists (PZM21 and SR-17018), unbiased morphine, and the β-arrestin-2-biased agonist, fentanyl. The study involved docking and molecular dynamics simulations carried out for three crystal structures of the target at a microsecond scale, followed by the statistical analysis of ligand–protein contacts. The interaction frequency between the modeled compounds and the subsequent residues of a protein during the simulation was also correlated with the output of in vitro and in vivo tests, resulting in the set of amino acids with the highest Pearson correlation coefficient values. Such indicated positions may serve as a guide for designing new G protein-biased ligands of the µ opioid receptor.

## 1. Introduction

Opioids are effective analgesics widely used for severe pain treatment. However, the majority of them, including morphine, produce side effects limiting their use, such as respiratory depression, constipation, and addiction. An epidemic in both western and developing countries of opioid use disorder and overdose deaths from prescription opioids has led recently to the search for new painkillers deprived of this action or with limited effects—in particular, respiratory depression. One of the most interesting approaches is based on the observation that an opioid acting via the μ opioid receptor may activate intracellular signal pathways with varying strength. These ideas of so-called biased signaling or functional selectivity suggest that some opioids may activate the G protein signal pathway and mediate the analgesic effect via the μ opioid receptor and avoid stimulation of the β-arrestin-2 pathway (which seems to be involved in the observed side effects [1]) at the same time. Therefore, the main interest in the case of the μ opioid receptor ligands is focused on the synthesis of opioid G protein-biased agonists that preferentially activate the G protein but not the β-arrestin-2 pathway. Identification of the crystal structure of μ opioid receptors has opened up, on the one hand, a better understanding of the structure of opioid receptors and, on the other, the development of new computer modeling technologies that have enabled the identification and synthesis of new biased opioids. One of them was PZM21, selected by molecular docking after virtual testing for selective bias activation of the μ opioid receptor [2]. This substance is in fact characterized by functional selectivity because it activates the G protein but not β-arrestins. In preclinical studies, PZM21 has been shown to be analgesic, with no conditioned place preference (CPP) production or locomotor stimulation observed [2,3]; on the other hand, it led to the development of rapid analgesic tolerance in the hot plate and tail-flick tests in mice [3,4] and caused respiratory depression [4]. Bohn’s group [5] recently identified a series of biased μ opioid receptor agonists. Among them, SR-17018 appears to be of great interest because it displays the highest bias towards G protein signaling compared to β-arrestin-2 recruitment [5] and shows antinociceptive activity and a very little respiratory suppression in mice [6]. In addition, long-term oral administration of SR-17018 did not lead to antinociceptive tolerance, prevented morphine withdrawal, and restored morphine antinociception in morphine-tolerant animals [6].

The emergence of new G protein-biased ligands has stimulated studies aimed at developing new ligands of this type, as well as research on the detailed mode of action of the already developed biased compounds. This research involves both experimental and computational studies of various types. In silico methods support the design and development of new drugs at different levels, starting from searching for drug candidates, via optimization of their activity and physicochemical properties, to support in the analysis of their efficiency after introduction to the market [7,8,9]. Computational approaches also help in understanding the mechanisms of compound action and simulate processes occurring during receptor activation [10,11,12].

Molecular modeling approaches use various data types to derive predictions of compound activity. Ligand-based methods use information only on the structure of compounds [13,14,15,16], whereas structure-based tools rely on the spatial orientation of atoms of the target protein and use docking to predict ligand fitting in the respective binding site [17,18]. One ligand–receptor complex returned by a docking program captures just one moment of the mutual orientation of a compound and protein. More computationally demanding but also much more informative method is molecular dynamics (MD), which enables simulation of the behavior of the modeled system (e.g., ligand–protein complex) in time [19,20].

In this study, we apply docking and MD simulations to explain the activity profiles of selected µ opioid receptor agents. We compare and analyze using molecular modeling methods, docking and MD simulations, four selected opioids targeting the μ opioid receptor: novel G protein-biased μ opioid receptor agonists, PZM21 and SR-17018 [2,5], unbiased morphine, and the β-arrestin-2-biased agonist, fentanyl. Extensive in silico examination of these ligands with reference to their μ opioid receptor activity involved docking to three μ opioid receptor crystal structures, MD simulations carried out at a microsecond scale (2 µs), and the statistical analysis of ligand–protein contacts during simulations. The interaction frequencies of a compound with the subsequent amino acids were confronted with the outcome of the selected in vitro and in vivo tests and positions with the highest correlation were identified. The set of such selected residues should be of particular interest when designing new G protein-biased μ opioid receptor agents. In addition, the orientation of W293^6x48^ (residue important for μ opioid receptor activation) during simulation was examined and confronted with the ligand activity profiles. The residue numbering provided in superscripts follows the GPCRdb [21] numbering scheme.

## 2. Results and Discussion

### 2.1. Comparison of Modeled Ligands and µ Opioid Receptor Crystal Structures

The modeled compounds were compared in terms of their structures and selected physicochemical properties, which were determined using InstantJChem [22] (Table 1).

The data gathered in Table 1 draw attention to the relatively high logP value of SR-17018 (4.75) and as many as eight rotatable bonds in PZM21 and six in fentanyl. The high number of possible bond rotations can lead to difficulties in obtaining stable conformation in the binding site (for comparison, there are no rotatable bonds in the morphine structure), whereas logP values affect the ability of a compound to penetrate the blood–brain barrier. Morphine, despite the rigid structure, is also characterized by very low logP (0.90) and the highest number of hydrogen bond acceptors: four.

In this study, we used three µ opioid receptor crystal structures—records with the following PDB codes were used: 4DKL [23], 5C1M [24], and 6DDF [25] (Table 2). The last two crystals refer to the active conformation of the µ opioid receptor, whereas 4DKL is co-crystallized with BF0, which acts as a µ opioid receptor antagonist and keeps the protein in its inactive form. Despite preserved protein activation occurring in 5C1M and 6DDF crystals, these two structures also differ from each other, as the former is activated via a small-molecule agonist, whereas 6DDF activation occurs via the highly selective peptide agonist for the µ opioid receptor, DAMGO. The peptide-like-based activation reflects the naturally occurring process of µ opioid receptor activation; however, the resolution (a very important factor in terms of the application of a particular structure in molecular modeling tasks) of 6DDF (3.5 Å) is significantly worse than the resolution of 5C1M (2.1 Å).

**Figure 1:**
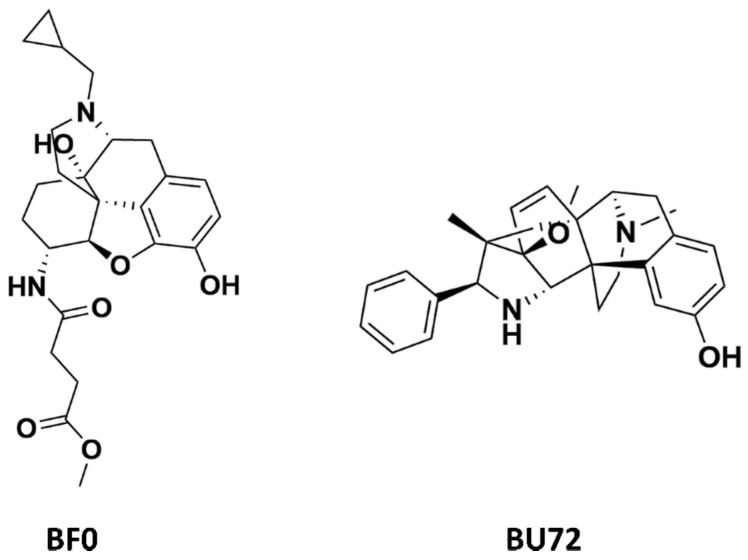
Small molecule ligands co-crystallized with µ opioid receptor crystal structures used in the study.

Due to high variation in the residue positions, resolution, and structure of co-crystallized ligands (also influencing the shape of the binding pockets, Figure 1), all calculations conducted within the study were carried out for all three µ opioid receptor crystals.

A comparison of the compound structures presented in Table 1 and Figure 1 indicates that the ligands co-crystallized with µ opioid receptor (BF0 and BU72) are significantly different from the analyzed compounds: PZM21, SR-17018, morphine and fentanyl. To describe these structure variations more formally, the Tanimoto coefficient [26] was calculated for each compound pair using InstantJChem at default settings (detailed numerical values are provided in Table 3). Its highest values were obtained for morphine, and they were equal to 0.764 and 0.595 for BF0 and BU72, respectively. Such high variation between modeled compounds and the co-crystallized ones might lead to changes in the protein structure during simulation with PZM21, SR-17018, morphine and fentanyl to adjust the protein binding site and form energetically favorable ligand–protein complexes.

### 2.2. Docking

**Figure 2:**
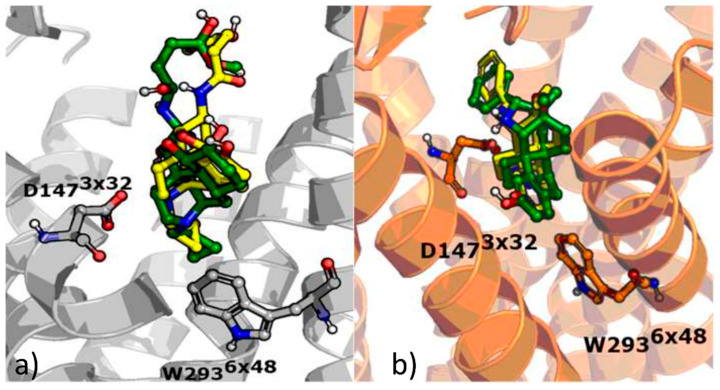
Results of the redocking experiments of co-crystallized ligands BF0 and BU72 to (**a**) 4DKL and (**b**) 5C1M crystal structures—green: co-crystallized conformation, yellow: ligand orientation obtained in docking.

Before docking the modeled compounds, the validity of the methodology was verified via redocking of BF0 and BU72 to their respective crystal structures. The comparison of the obtained docking poses with crystallized compound conformations is presented in Figure 2. As docking enabled us to obtain orientations similar to the co-crystallized compound fitting (RMSD values between the co-crystallized and docked pose were equal to 1.36 Å and 0.34 Å for BF0, and BU72, respectively), the analogous approach was used for modeling of PZM21, SR-17018, morphine and fentanyl.

**Figure 3:**
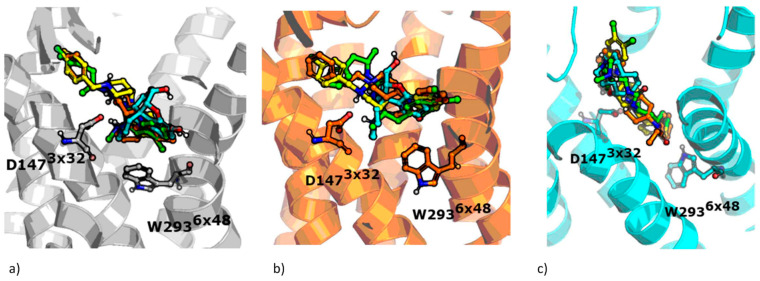
Docking poses of examined compounds at µ opioid receptor crystal structures, (**a**) 4DKL, (**b**) 5C1M, (**c**) 6DDF. PZM21: green; SR-17018: yellow; morphine: cyan; fentanyl: orange. GPCRdb numbering scheme for amino acid labeling is used. Coloring of particular crystal structures is consistent through the whole manuscript.

**Figure 4:**
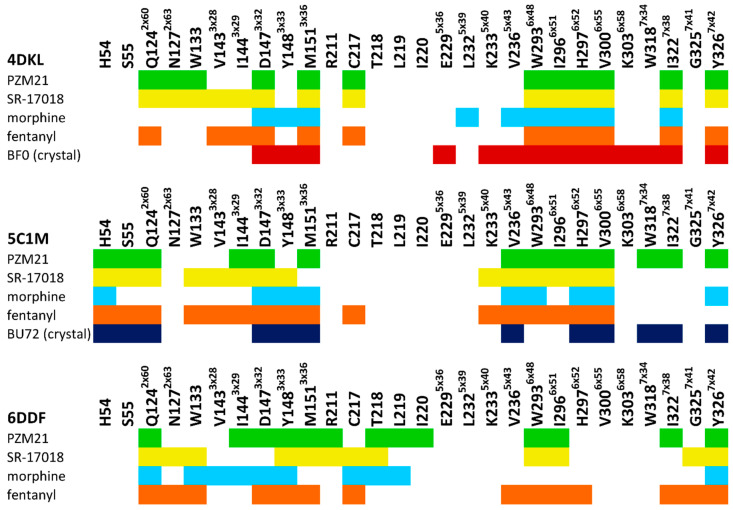
Ligand–protein contacts occurring within complexes obtained in docking to various µ opioid receptor crystal structures. For comparison, interactions of co-crystallized BF0 and BU72 with respective crystal structures are included.

General compound orientations in the µ opioid receptor binding site for different crystals are depicted in Figure 3 and a more detailed analysis of ligand–protein contacts in the form of the interaction matrix is presented in Figure 4.

Figure 3 clearly indicates that all the compounds occupy the same region of the binding site, although, due to the structural differences, much less space is taken up by morphine than the other ligands considered. As shown in Figure 4, all ligands consistently make contact with D147^3x32^ and the sixth transmembrane helix (TM6) of the µ opioid receptor for the 4DKL and 5C1M crystal structures. These observations are consistent with the very recent work of Zhao et al. [27], where docking and MD simulations were carried out for PZM21, TRV130 (oliceridine), and morphine using one crystal structure (5C1M) to explain the differences in their functional profiles. On the other hand, the docking poses obtained for 6DDF did not make such frequent contact with TM6—fentanyl made contact with three residues from this protein region, and PZM21 and SR-17018 interacted only with W293^6×48^ and I296^6×51.^ However, morphine lacked any contact with TM6. Instead, morphine made contact with five amino acids from TM3 when docked to 6DDF, whereas for 4DKL and 5C1M, it interacted only with D147^3×32^, Y148^3×33^, and M151^3×36^ from TM3. For 6DDF-based docking, all the ligands also interacted with Q124^2×60^, which is unique for this crystal structure (for 4DKL and 5C1M, contact with this residue is missing for morphine). PZM21 and SR-17018 also interacted with more amino acids from TM2 (in comparison to morphine) for 4DKL- and 5C1M-based dockings.

The studied compounds shared interaction patterns presented by Zhao et al., especially in terms of contacts with the set of key residues: D147^3×32^, Y148^3×33^, and H297^6×52^. However, although the compounds occupied the same region of the binding site and were oriented similarly in the binding pockets both in our study and in Zhao’s research, there were also some contacts that we did not observe in our poses. For example, morphine lacked contacts with Q124^2×60^ and I144^3×29^, K303^6×58^ and W318^7×34^, and PZM21 did not interact with W133 or Y148^3×33^.

The obtained interaction patterns were also confronted with the contact networks formed by the co-crystallized ligands. Antagonist BF0, present in the 4DKL crystal, similarly to modeled compounds, interacted with D147^3×32^, M151^3×36^, and a collection of amino acids from TM6. In contrast, BF0 did not come into contact with any residue from TM2 but made interactions with several residues from TM5 (unlike morphine, PZM21, SR-17018, and fentanyl). Contact patterns of the co-crystallized agonist BU72 are more similar to the modeled compounds. BU72 interacted with H54, S55, and Q124^2×60^ (as for all examined compounds, except morphine), and it possessed the same interaction network with TM3 and TM5 as morphine and with TM7 as PZM21. BU72 also contacted H297^6x52^, and V300^6×55^ from TM6, but it did not form an interaction with W293^6×48^.

The variations in the results obtained in different studies and for different crystal structures indicate the necessity of extending the number of protein structures (either crystal structures or homology models) used in molecular modeling tasks, e.g., during virtual screening. The co-crystallized ligand implies the conformation of the binding site; therefore, the output of docking studies performed for the rigid protein is biased due to the protein adjustment to a co-crystallized agent. The recommendation of using more than one receptor conformation for docking studies was also indicated by Mordalski et al. [28] in the case study of beta-adrenergic receptor type 2.

### 2.3. Molecular Dynamics and Correlation Studies

As docking captures only one particular moment of interaction between a ligand and protein, it cannot fully explain dependencies between compound behavior towards a given target in relation to its docking pose. The more poses considered, the higher the amount of information provided. To ensure study comprehensiveness, extensive MD simulations with a length of 2000 ns were carried out for each compound–crystal structure combination (12 simulations were run in total). The relative total number of 1000 frames was produced from each simulation, giving a picture of 1000 ligand–protein mutual orientations.

**Figure 5:**
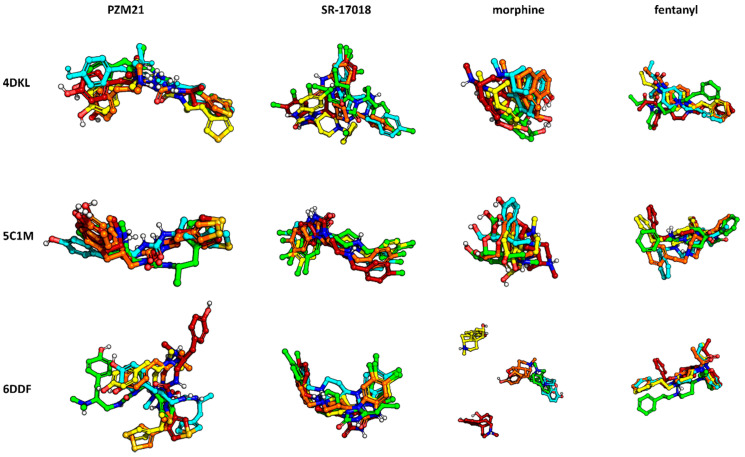
Stability of compound orientations in the binding site during simulations. Green: starting pose; cyan: 250th frame; orange: 500th frame; yellow: 750th frame; red: 1000th frame.

**Figure 6:**
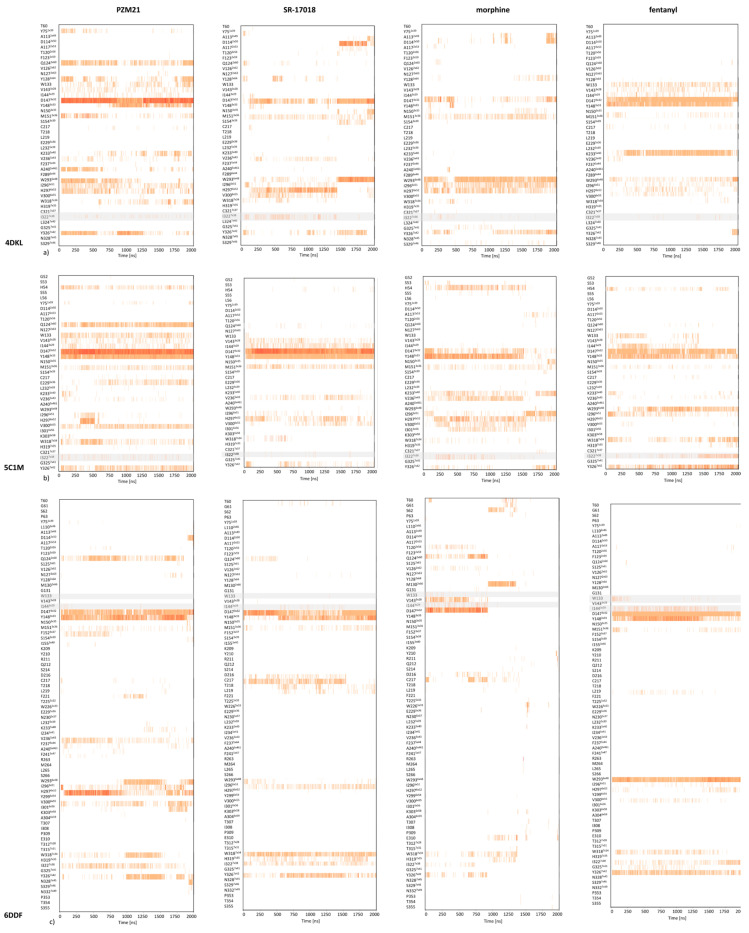
Ligand–protein contacts occurring during molecular dynamics simulations with (**a**) 4DKL, (**b**) 5C1M, and (**c**) 6DDF µ opioid receptor crystal structure. Positions for which the highest correlation between the output of the tail flick experiment and interaction frequency were indicated are shaded.

The MD results were analyzed from two perspectives: the stability of compound orientations in the binding site (Figure 5) and changes in ligand–protein contacts that occurred in time (Figure 6).

Figure 5 indicates that all of the compounds were most stable when simulated with the 5C1M crystal structure. PZM21 simulated in 5C1M slightly changed its initial position at the beginning and then remained in the adopted conformation. PZM21 in 4DKL was oscillating around its initial pose; however, when 6DDF was taken for simulation, the PZM21 pose varied a lot during the whole simulation time, and the PZM21–6DDF combination resulted in the least stable compound conformation out of all analyzed setups. The highest variation in the compound pose in the binding site was observed for morphine in the 6DDF-based simulation, which changed its initial position and left the binding cavity during the simulation course (after approximately 1000 ns). Fentanyl simulated with 4DKL and 6DDF moved at the beginning of the simulation from its initial position; then, it kept its pose for at least 1500 ns, to slightly change again the conformation when the simulation length approached 2000 ns. In 5C1M-based MDs, fentanyl did not move from the initially occupied region of the binding pocket and the mass center of the compound remained in the same area, with its conformation varying during the whole simulation.

Analyzing the contact patterns presented in Figure 6, one can see that the characteristic MD simulation output is the very strong interaction of PZM21 with D147^3×32^, even during the simulation with 6DDF, where the compound orientation was in general very unstable (analogous observation also reported by Zhao et al. [27]). Many research studies have indicated this residue as very important for activity towards the µ opioid receptor [29,30]. PZM21 also made consequent contact with Y148^3x33^, although when simulated with 4DKL, the interaction was formed after approximately 750 ns of simulation. PZM21 changed its orientation very frequently during simulation with 6DDF; however, with D147^3×32^ and Y148^3×33^, contact was present during the whole simulation. There was also a set of residues with which it interacted periodically, such as F221, W293^6×48^, W318^7×34^, and Y326^7×42^ (the most frequent contacts between 1000 and 1500 ns of the simulation).

The orientation of PZM21 within the 5C1M binding site was very stable; nevertheless, even in the case of this setup, short contact (occurring only between 250 and 500 ns) with I296^6×51^ and H297^6×52^ was observed. Within this period, the intensity of interaction with W318^7×34^ also strongly increased.

Consistent contact with D147^3×32^ is also visible for fentanyl (for all crystal structures), although the interaction is not so strong, as it is in the case of PZM21. This compound also gains interaction with W293^6×48^ in the simulation with 6DDF, despite its initial lack (the contact occurs constantly during the whole simulation). Fentanyl–W293^6×48^ contact is also present in MDs with 4DKL and 5C1M, although in the former case, the interactions are relatively sparse.

The last 500 ns of simulation of SR-17018 with 4DKL resulted in the formation of ligand contact with D114^2×50^, W293^6×48^, Y326^7×42^, and an increase in the contact frequency with D147^3×32^. At the same time, it stopped making contact with V236^5×43^, I296^6×51^, H297^6×52^, V300^6×55^, and I322^7×38^. On the other hand, the simulation of SR-17018 with 5C1M and 6DDF resulted in a very consistent and stable interaction pattern over time. Only for 6DDF, an increase in contact intensity with Y148^3×33^ after 500 ns of simulation and loss of contact with D216 and C217 during the last 500 ns of the simulation were observed. Interestingly, morphine displayed a very unstable conformation when simulated with 6DDF; despite the strong interaction with D147^3×32^, the contact was lost halfway through the simulation course. In the simulations with 4DKL and 5C1M crystal structures, the contact with D147^3×32^ occurred during the whole simulation time; however, it was not as frequent and strong as it was in the case of PZM21 and SR-17018.

Strong interaction of the modeled compounds with D147^3×32^ was also reported by Zhao et al. [27]. This finding is consistent for simulations carried out for all crystal structures. However, there is another observation indicated by Zhao et al., which led to different conclusions when various crystals were considered. Zhao et al. indicated the formation of a strong interaction of morphine with H297^6×52^, related to its shift deeper into the pocket during the simulation (this effect was not observed for PZM21). Similarly, in our simulations with 5C1M (crystal structure used by Zhao et al.), morphine made contact with H297^6×52^ after ~200 ns of simulation, which was maintained consistently until the end of the simulation by ~1800 ns. On the other hand, PZM21 also came into short contact with this residue (~300–500 ns), but then the interaction was lost and the compound did not interact with H297^6×52^, as reported by Zhao et al. [27].

However, similar to the docking studies, MD simulation output also varied depending on the crystal structure, as already discussed. Taking into account the described above interaction with H297^6×52^, the contact formation with this residue by morphine and its lack for PZM21 was observed only for simulations with 5C1M. For 6DDF-based studies, the situation is reversed, as morphine did not make contact with H297^6×52^ during the whole simulation, but PZM21 started to interact with this position after ~50 ns of simulation, and the contact was very intense up to ~1000 ns; then, although the interaction was not so strong, it continued until 2000 ns, when the simulation finished. In contrast, in the 4DKL-based studies, both morphine and PZM21 came into contact with H297^6×52^. For comparison, SR-17018 did not interact with H297^6×52^ at all for 6DDF; with 5C1M, it continuously came into contact with this residue (although the contact intensity was not very strong), and for 4DKL, the interaction was quite strong at the beginning of the simulation, but after ~1500 ns, the compound changed its conformation and the contact was lost.

These variations in the interaction patterns obtained for different crystal structures, as well as the changes in compound poses after relatively long simulation times (e.g., SR-17018 in 4DKL, which adopted a new pose after 1500 ns), confirm the necessity of applying more protein conformations in structure-based studies and indicate that the simulations should be as long as allowed by computational resources, as some events might not be observed in shorter dynamics.

The interaction schemes obtained in each MD simulation were confronted with selected experimental data produced on examined compounds (see [3,5,31] data gathered in Supporting Information Table S1) to indicate positions that should attract particular attention when developing new ligands of particular activity profiles. This was done by encoding the interactions occurring in each frame from the MD simulation in the form of interaction fingerprints (IFPs) [32] and calculating the Pearson correlation coefficient [33]. The correlation was calculated between the total number of contacts by the particular ligand with a given residue and the experimental parameter value, i.e., the activation of Galphai2 protein (Gai2 activation), activation of G protein-coupled inwardly rectifying (GIRK) potassium channels, recruitment of β-arrestin-2 (bArr2 recruitment), and G protein-activated inward rectifier potassium channel 2 (GIRK2 recruitment). In addition, Galphai-mediated cAMP inhibition value (cAMP inhibition) and efficacy of trafficking of µ opioid receptor to Rab-5 positive endosomes (Rab5 trafficking) were utilized.

**Figure 7:**
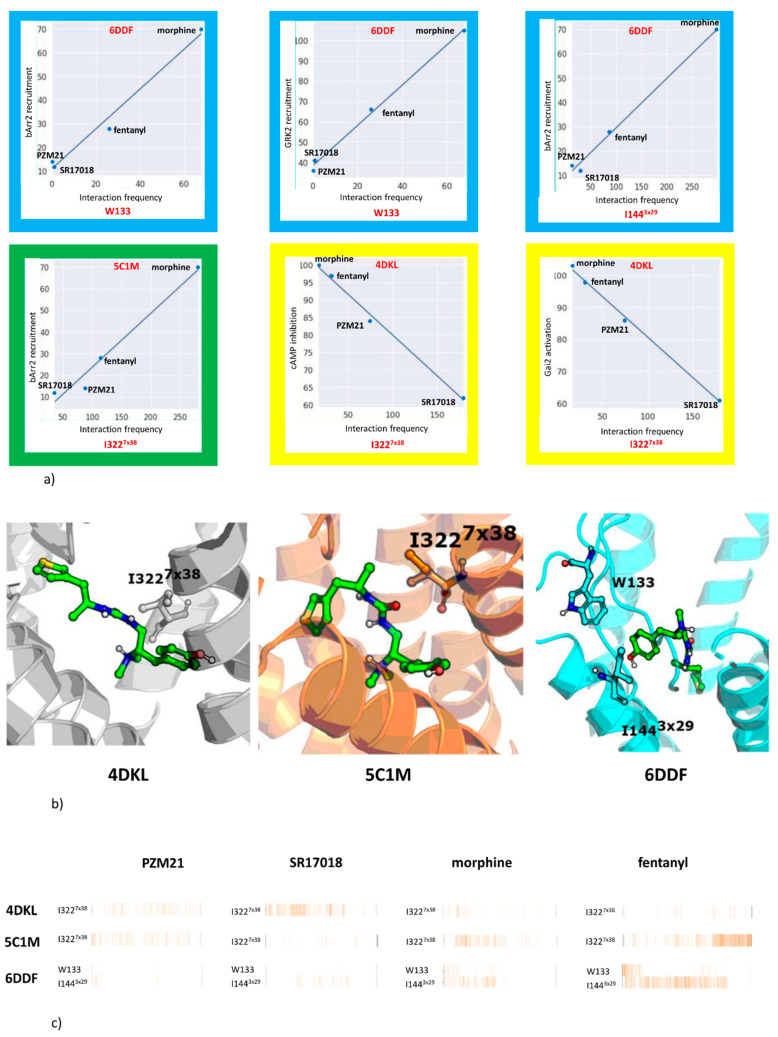
(**a**) Correlation charts between the interaction frequency and outcome of the in vitro experiments (cases with the highest correlation are presented with the Pearson correlation coefficients above 0.98) with frames’ color referring to particular crystal structure—yellow: 4DKL, green: 5C1M, blue: 6DDF; (**b**) indication of residues with the highest correlation presented in (**a**), (**c**) ligand–protein interaction diagrams obtained during MD simulations for residues presented in (**a**).

The outcome of the studies investigating the correlation between the contact frequency between ligand and a particular amino acid and the outcome of experimental tests is presented in Figure 7 and in Table 4. Cases with the highest values of the Pearson coefficient are presented in the figure, and the remaining highly correlated cases are included in Table 4 (respective charts are presented in the Supporting Information, Figure S1). Frames around each separate chart indicate the crystal structure for which the particular correlation was examined.

Out of the six presented charts with the highest experiment–contact pattern correlations, three were related to simulations with 6DDF, and in the case of three of them, high correlations were found for the β-arrestin-2 recruitment assay. The list of highly correlated amino acids was composed of just one residue for 5C1M and 4DKL (I322^7×38^). For the former crystal, it was obtained in the β-arrestin-2 recruitment experiment, which led to a Pearson correlation coefficient above 0.98 (it was equal to 0.985), whereas for 4DKL, it was cAMP inhibition and Gai2 activation experiments, which were highly related to the interaction frequency of compounds with I322^7×38^ during MD simulations (Pearson correlation coefficients were equal to 0.997 and 0.998, respectively). Moreover, 6DDF-based correlations indicated amino acids from different regions of the protein, W133 and I144^3×29^, with the former related to the β-arrestin-2 recruitment assay and experiments based on GRK2 recruitment. 

The results indicate that despite extensive simulation time (2 µs) enabling significant changes in the protein structure, the initial conformation forced by the co-crystallized ligand influenced the results during the whole simulation time. The correlation coefficients between the ligand–contact frequency and the outcome of experimental studies vary significantly for a particular crystal structure. The highest number of experiments with any “highly correlated” residue occurred for 6DDF.

**Figure 8:**
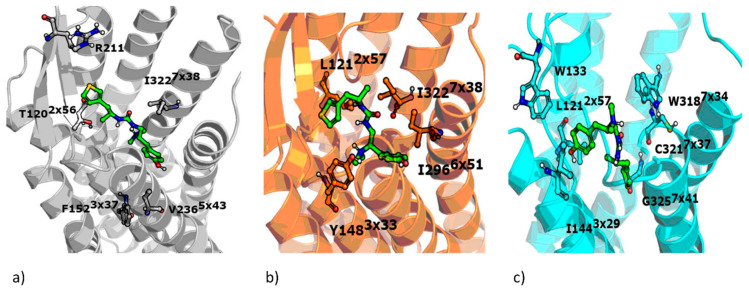
Residues with high correlation between the interaction frequency and experimental tests output obtained for (**a**) 4DKL, (**b**) 5C1M, and (**c**) 6DDF. PZM21 visualized for reference.

The positions of residues gathered in Table 4 are visualized in the respective crystal structures in Figure 8.

When the unbiased morphine is confronted with biased agonists examined in the study, it appears that, in general, morphine interacts less intensively with D147^3×32^, which is consistently observed for all crystal structures, but this is most visible for 5C1M-based simulations. On the other hand, in 5C1M-based simulations, morphine interacted more frequently with K333^5×40^, V236^5×43^, and I301^6×56^. Simulations with 4DKL revealed more intense interaction of morphine with W293^6×48^ in comparison to biased ligands, whereas with 6DDF-based studies, PZM21 and SR-17018 interacted more frequently with I296^6×54^, I322^7×38^, and Y326^7×42^. These abovementioned residues should be monitored; however, their discrimination potency between biased and unbiased ligands should be verified for a higher number of compounds, with each activity profile considered.

Zhao et al. indicated W293^6×48^, W318^7×35^, Y326^7×42^, and Y336^7×53^ as residues that affect the receptor function [27]. These positions were not indicated in our statistical analyses; however, it should be pointed out that the method of residue indication in our study differed from Zhao’s approach. We focus on the compound contacts with the protein and analyze its behavior in the binding site, whereas Zhao et al. examined relative positions of particular amino acids and on this basis discussed the role of particular amino acids with reference to the activity profile of examined compounds. Therefore, the conclusions drawn from MD simulations in our study and those obtained by Zhao et al. should be compared cautiously and considering the above-described methodological differences.

**Figure 9:**
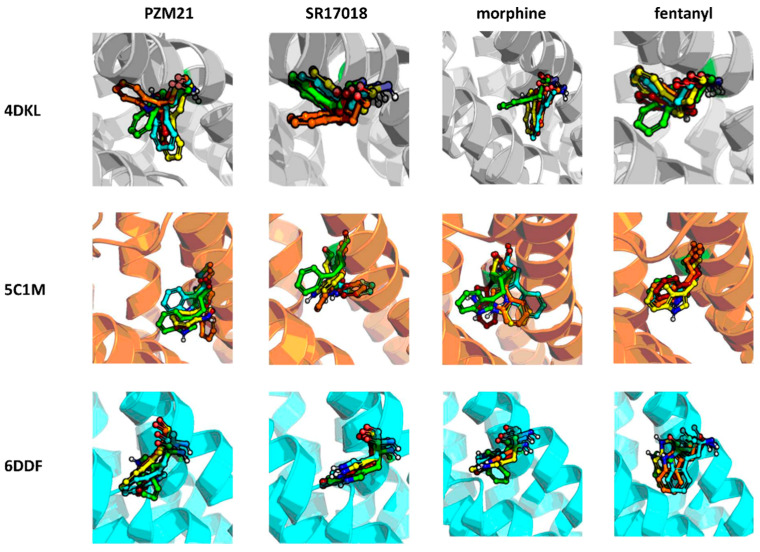
Position of W2936x48 during simulations with various ligands—green: starting pose; cyan: 250th frame; orange: 500th frame; yellow: 750th frame; red: 1000th frame.

To examine the influence of the modeled ligand, as well as to correlate results obtained in the MD simulations with functional activity of the µ opioid receptor, an analysis of the position of W293^6×48^ (residue important for the activation of opioid receptors [27]) for various setups was carried out (Figure 9). Its conformational changes imply the arrangement of the fifth and sixth transmembrane helices (TM5 and TM6), transmitting a signal to another protein region (intracellular loop).

The results show that the W293^6×48^ position changed during the simulations in the majority of cases. When the µ opioid receptor was in its inactive conformation (4DKL crystal), the protein simulation with the agonistic agents resulted in changes in its orientation. Interestingly, the direction of changes varies depending on the ligand. For morphine, W293^6×48^ moved lower, towards the inner part of the receptor, whereas for fentanyl and SR-17018, it shifted towards the extracellular part of the protein. These events took place at the beginning of the simulations and the residue orientation remained the same until the end of the simulation. PZM21 initially induced a similar change in W293^6×48^ position as morphine; however, in the middle of the simulation, the residue adopted a similar orientation for a while as in the case of SR-17018 and fentanyl. The activated form of the receptor present in the 5C1M crystal structure also led to variation of the W293^6×48^ position during simulation: in this case, morphine and SR-17018 led to similar changes in the residue position, and for PZM21, variation in the W293^6×48^ position occurred which was similar to its simulation with 4DKL. Interestingly, the W293^6×48^ position did not change during simulation with fentanyl. In simulations fentanyl–6DDF and SR-17018–6DDF, a consistent position of W293^6×48^ was also observed. Morphine simulated with 6DDF slightly changed its orientation at the beginning of the simulation towards the extracellular part of the receptor, and the PZM21–6DDF simulation resulted only in a minimal change in W293^6×48^ orientation.

## 3. Materials and Methods

The compounds were prepared for docking using LigPrep [34] from the Schrödinger Suite: protonation states were generated at pH 7.4+/−0.0, and all possible stereoisomers were enumerated; other settings remained at default. The crystal structures used in the study were fetched from the PDB database [35] and prepared for docking using the Protein Preparation Wizard from the Schrödinger Suite. Mass center of the co-crystallized ligand constituted the grid center in each case, and the grid size was set to 23 Å. The docking was carried out in Glide [36] in extra precision mode. The obtained ligand–receptor complexes with the best docking score constituted an input for MD simulations. They were carried out in Desmond [37], using the TIP3P solvent model [38], POPC (palmitoyl-oleil-phosphatidylcoline) as a membrane model, the OPLS3e force-field under the pressure of 1.01325 bar, and a temperature of 300 K. The box shape was orthorhombic, with a size of +10 Å × +10 Å × +10 Å. In each case, the system was neutralized by addition of the appropriate number of Cl- ions and relaxed before simulation; the duration of each simulation was equal to 2000 ns. The interactions between ligands and the respective proteins during MD simulations were analyzed using Simulation Interaction Diagram from the Schrödinger Suite. Interactions occurring in each frame of the performed simulations were encoded in the form of interaction fingerprints (IFPs) [29]. Then, for each nonzero column, the Pearson correlation coefficient between the total number of contacts formed with a particular ligand by a given residue with the experimental parameter value was determined.

## 4. Conclusions

In this study, in silico examination of the activity profiles of selected µ opioid receptor agents (PZM21, SR-17018, morphine, and fentanyl) was carried out. Three crystal structures of the target were used for docking and MD simulations and the obtained ligand–protein interaction patterns were confronted with the outcome of selected experimental tests. The variation in the obtained results clearly indicates the necessity of using as much structural data as possible in structure-based studies and not focusing on one receptor conformation. Each computational setup provided new insight into the given problem and indicated a new set of amino acids that should be taken into account when examining compound interaction profiles in silico. Moreover, the study enabled the indication of amino acids that should attract special attention when designing new ligands with particular properties: W133, I144^3×29^, I322^7×38^. Apart from these residues, there were over a dozen positions correlated with other experimental output, for single crystal structures, which also should be taken into account during µ opioid receptor ligand modeling.

## Figures and Tables

**Table 1:** Structure and properties of the µ opioid receptor ligands examined in the study.

Compound Symbol	Compound Structure	µ Opioid Receptor Activity	Molecular Weight *	LogP **	# H Bond Acceptors	# H Bond Donors	# Rotatable Bonds
PZM21	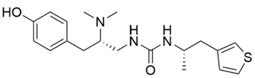	G protein-biased agonist	361.50	2.85	3	3	8
SR-17018	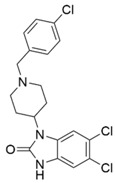	G protein-biased agonist	410.73	4.75	2	1	3
morphine	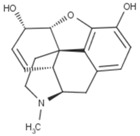	Unbiased agonist	285.34	0.90	4	2	0
fentanyl	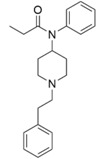	β-arrestin-2-biased agonist	336.47	3.82	2	0	6

* calculated for base compound form; ** determined in InstantJChem. **#** meaning ‘the number of’.

**Table 2:** Summary of characteristics of crystal structures used in the study.

PDB ID	Receptor State	Resolution (Å)	Co-Crystallized Ligand
4DKL	inactive	2.8	BF0 (Antagonist)
5C1M	active	2.1	BU72 (Agonist)
6DDF	active	3.5	DAMGO (Peptide agonist)

**Table 3:** Tanimoto coefficient values between the co-crystallized ligands and the examined compounds.

Modeled Ligand	BF0	BU72
PZM21	0.306	0.318
SR-17018	0.297	0.331
morphine	0.764	0.595
fentanyl	0.289	0.322

**Table 4:** Amino acids with the highest values of the Pearson correlation coefficient (above 0.94) between the ligand–residue contact frequency and experimental value (data produced on the basis of the pEC_50_ values of selected agonists for some of the pathways measured at the µ opioid receptor gathered in Gillis et al. [31]).

Crystal Structure/Parameter.	Gai2 Activation	cAMP Inhibition	bArr2 Recruitment	Rab5 Trafficking	GIRK Activation	GRK2 Recruitment
4DKL	T120^2×56^, I322^7×38^	T120^2×56^, V236^5×43^	F152^3×37^, R211			
5C1M		I296^6×51^	L121^2×57^, I322^7×38^	Y148^3×33^		I322^7×38^
6DDF	W318^7×34^	W318^7×34^	L121^2×57^, W133, I144^3×29^, C321^7×37^, G325^7×41^	W133		W133, I144^3×29^

## Supplementary Materials

The following are available online. Table S1. In vitro and in vivo data used in the study (expressed in the form of pEC_50_ values). Figure S1. Correlation charts between the interaction frequency with a particular residue and outcome of experimental tests.
