# Supplementary material for: Molecular Modeling of µ Opioid Receptor Ligands with Various Functional Properties: PZM21, SR-17018, Morphine, and Fentanyl—Simulated Interaction Patterns Confronted with Experimental Data
Source: Molecules. 2020 Oct 12;25(20):4636. doi: 10.3390/molecules25204636 (PMC7594085; doi:10.3390/molecules25204636)
Supplement: Supplementary file 1 [file molecules-25-04636-s001.zip › Table_S1.pdf]

Table S1. In vitro and in vivo data used in the study (expressed in the form of the pEC50 values).

**Compound/parameter**	**Gai2 activation**	**cAMP inhibition**	**bArr2 recruitment**	**Rab5 trafficking**	**GIRK activation**	**GRK2 recruitment**
PZM21	103	100	70	88	95	105
SR17018	98	97	28	75	88	66
Morphine	86	84	14	51	86	36
Fentanyl	61	62	12	59	78	41
