# Supplementary figures and images for: Molecular Modeling of µ Opioid Receptor Ligands with Various Functional Properties: PZM21, SR-17018, Morphine, and Fentanyl—Simulated Interaction Patterns Confronted with Experimental Data
Source: Molecules. 2020 Oct 12;25(20):4636. doi: 10.3390/molecules25204636 (PMC7594085; doi:10.3390/molecules25204636)

Gai2 activation

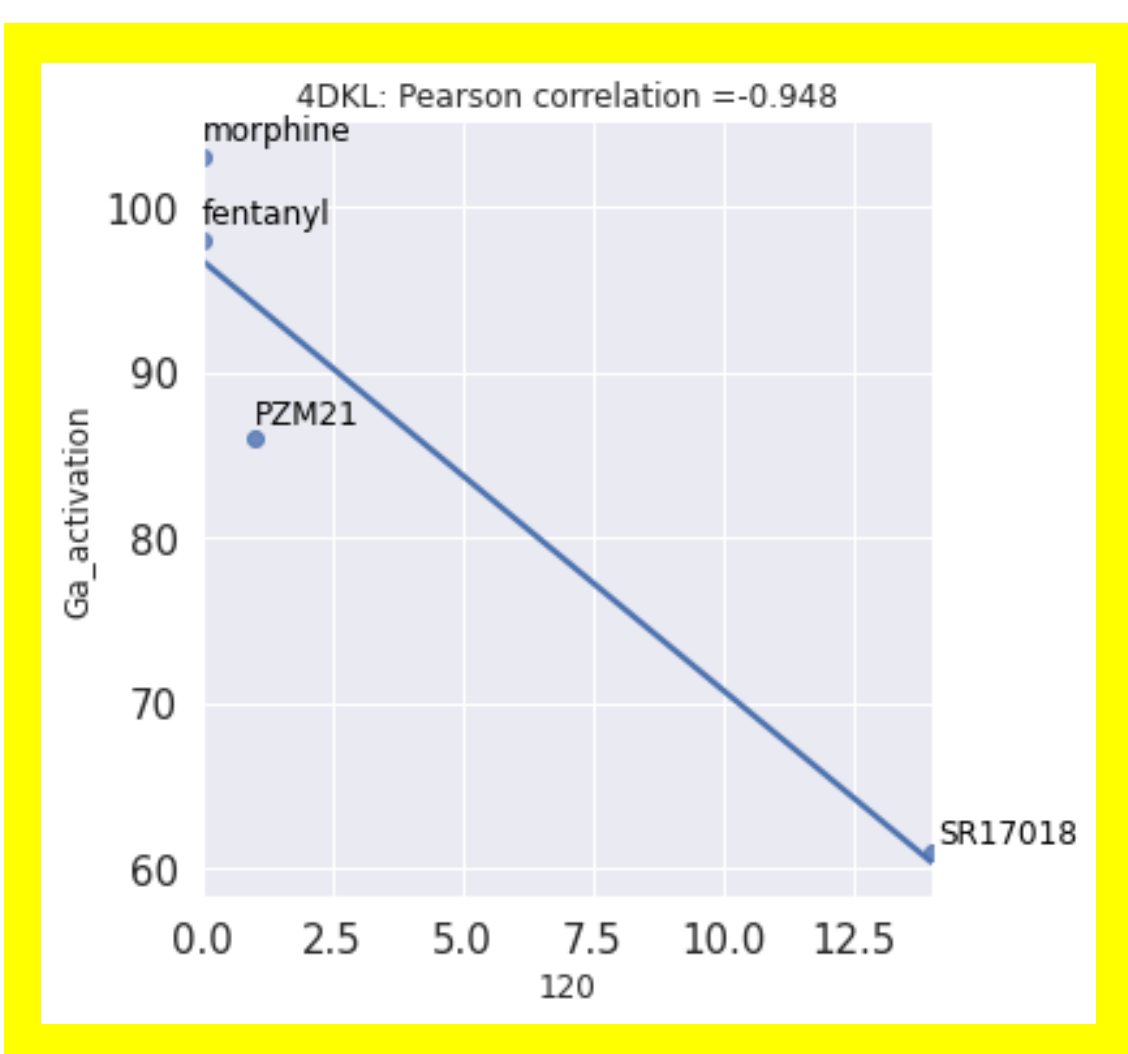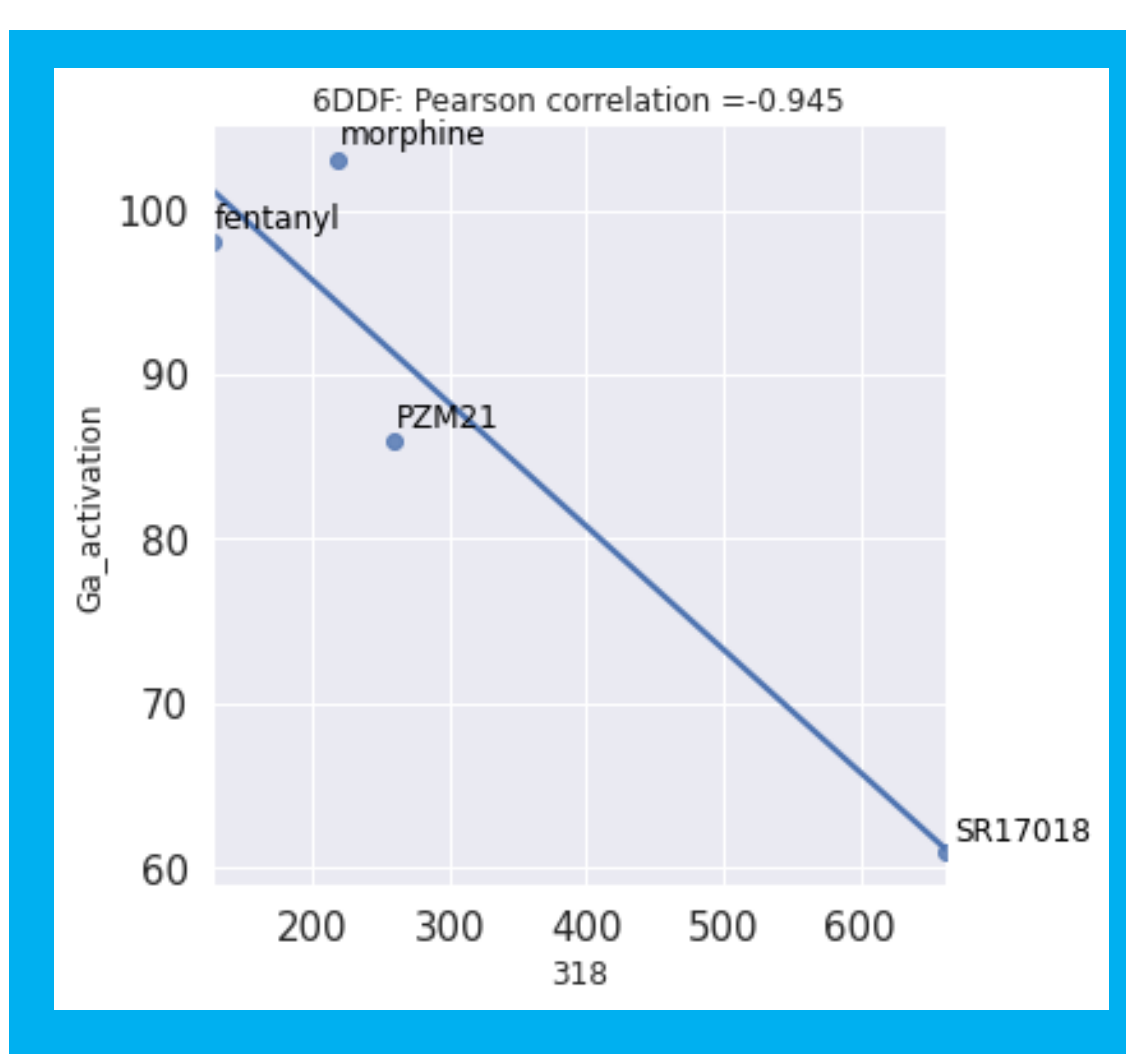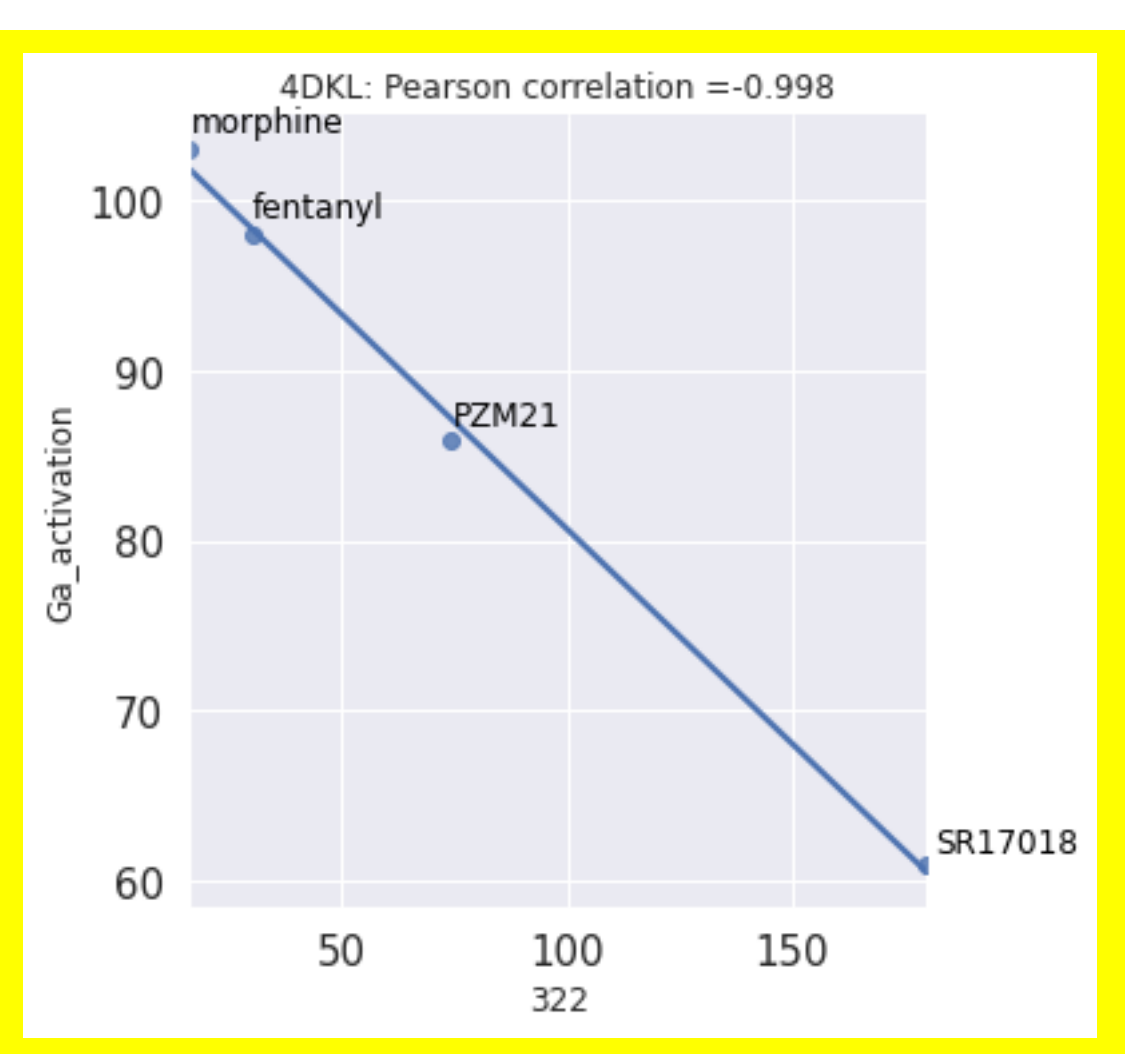

cAMP inhibition

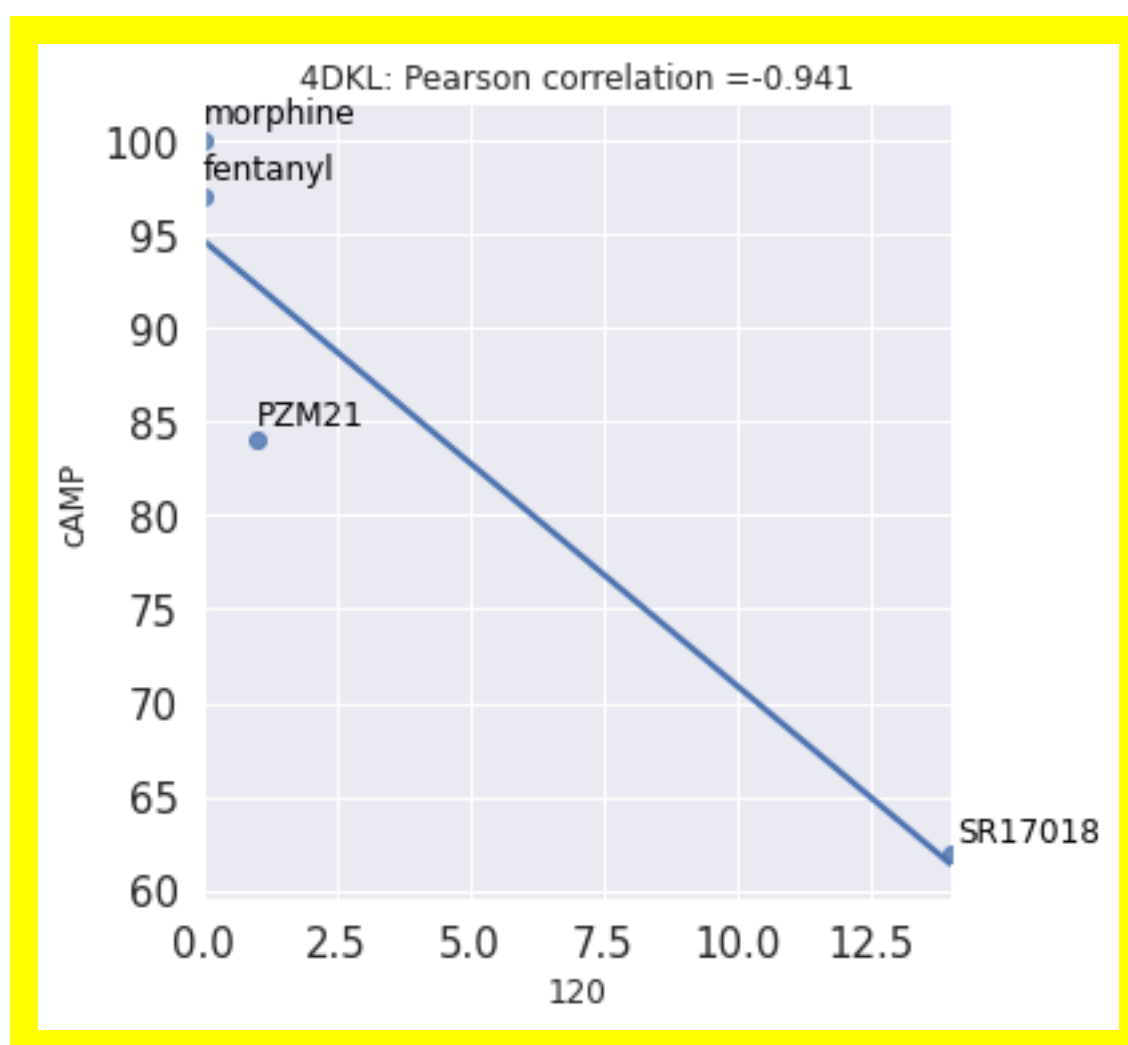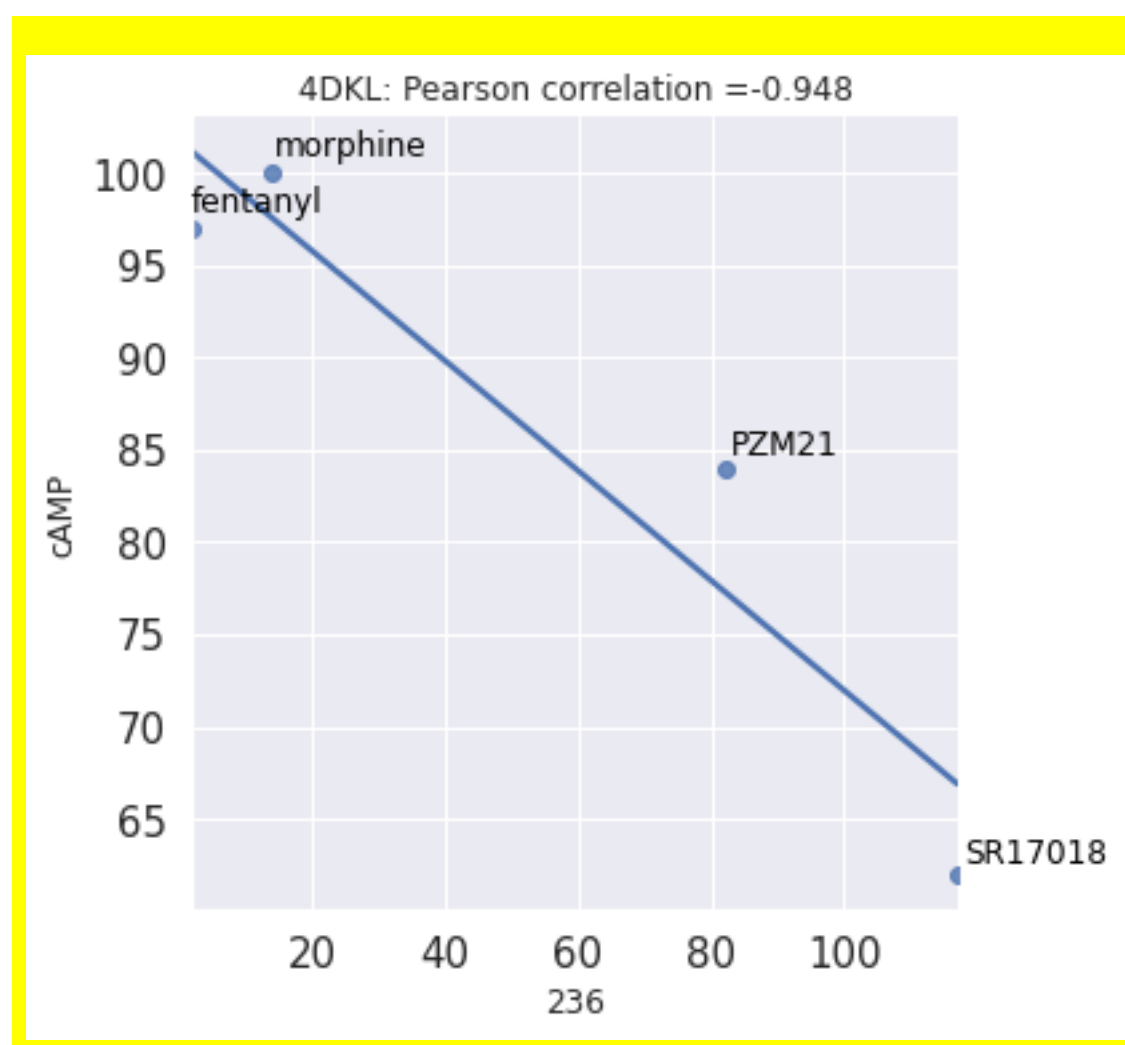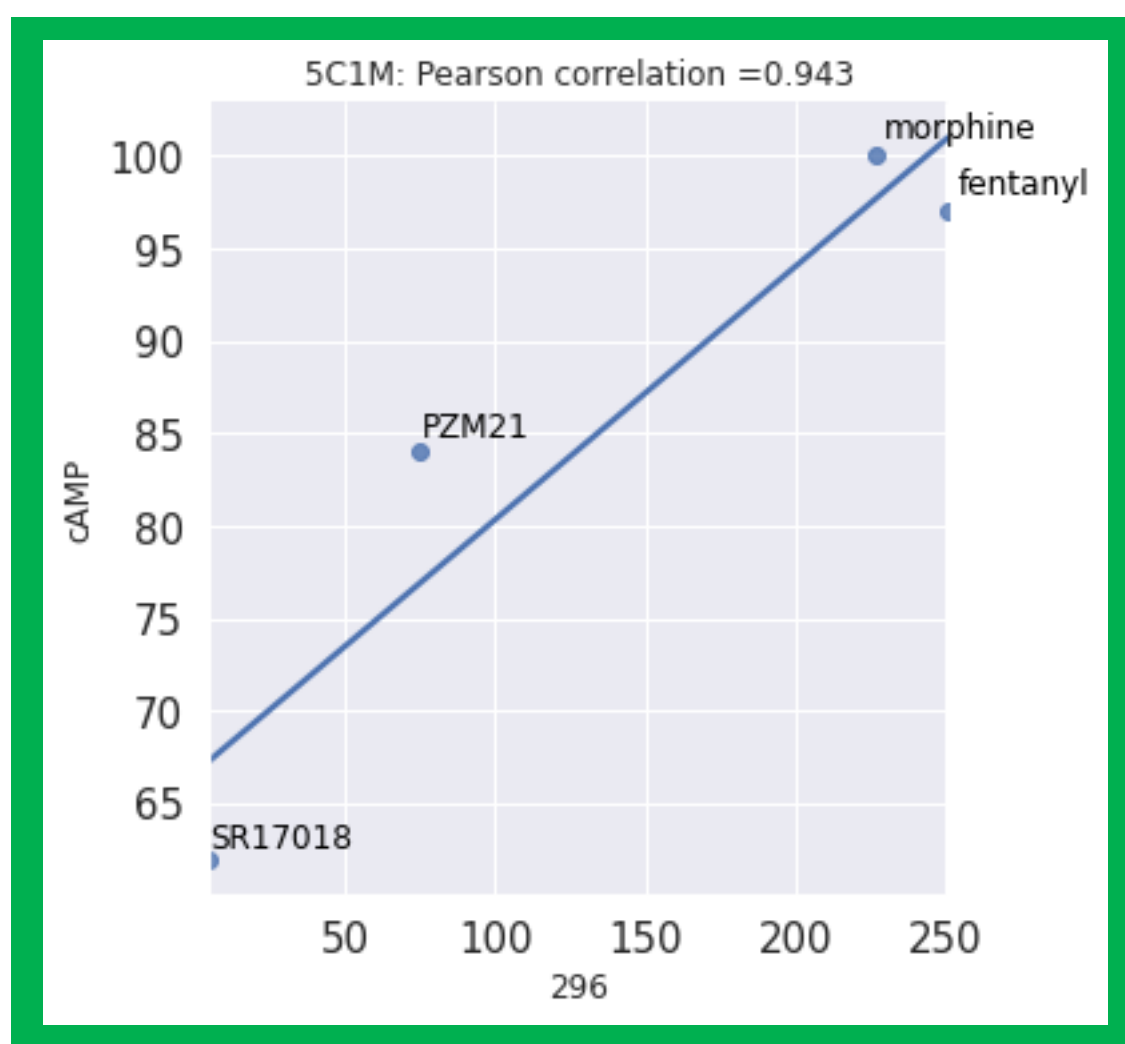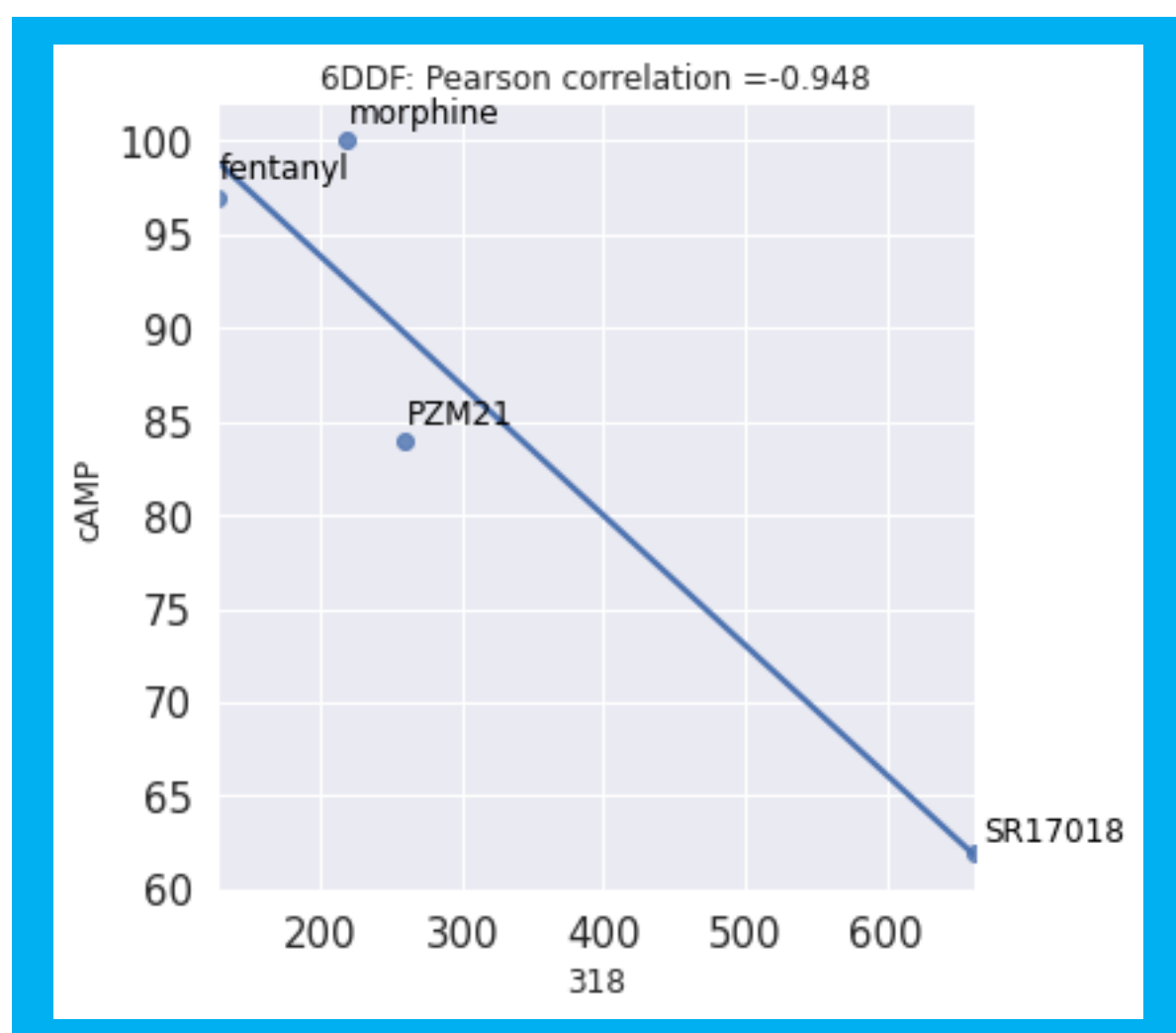

bArr2 recruitment

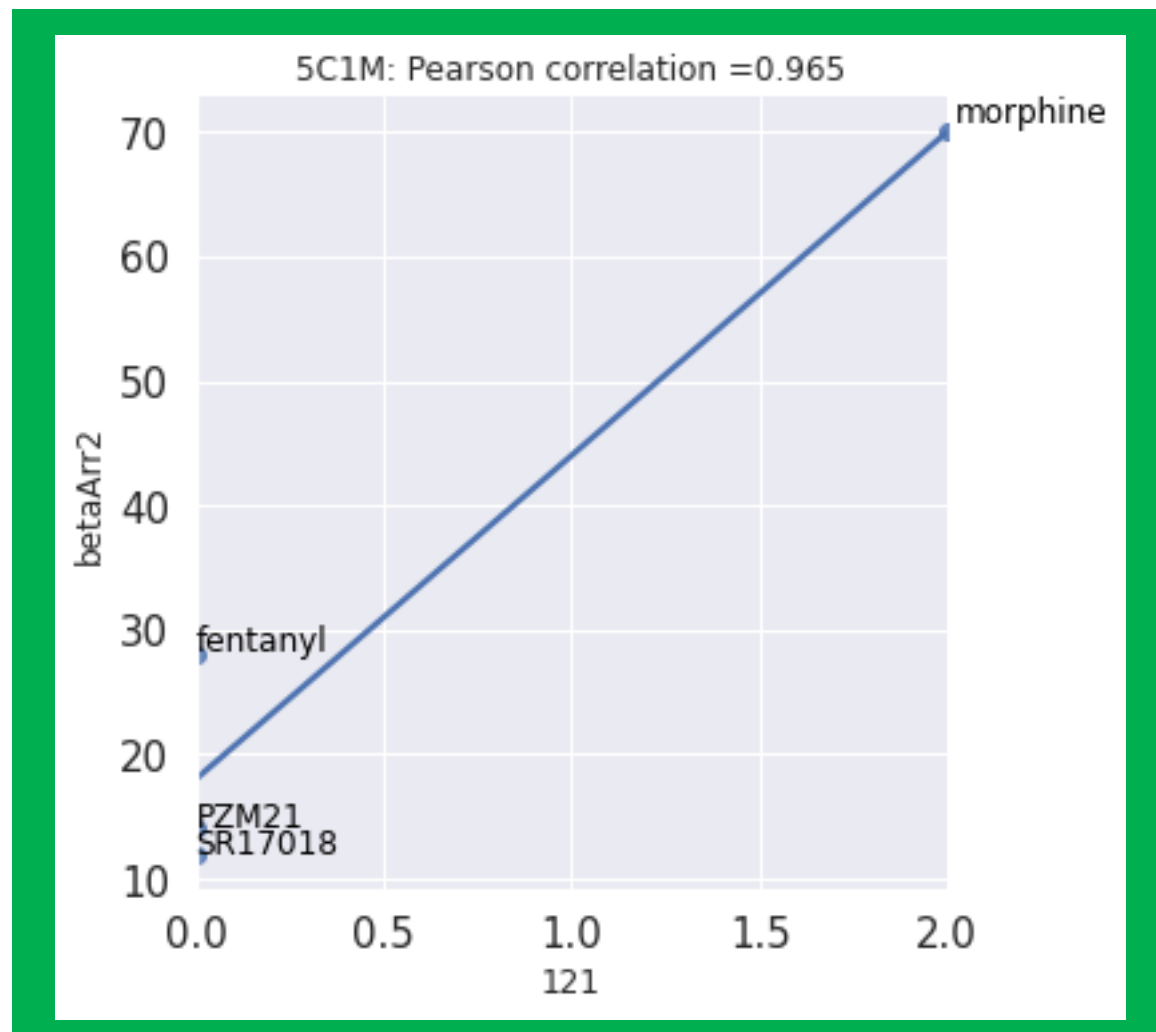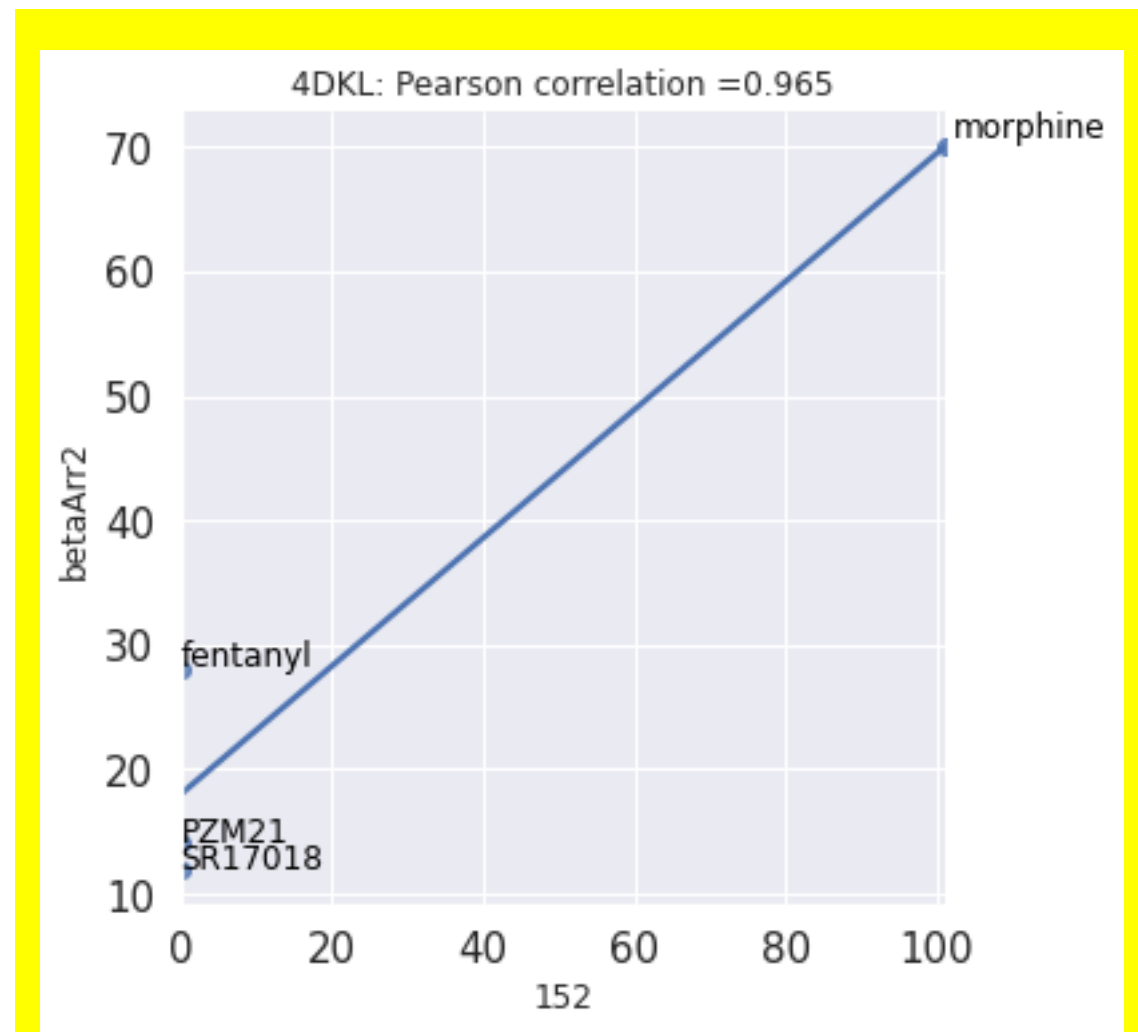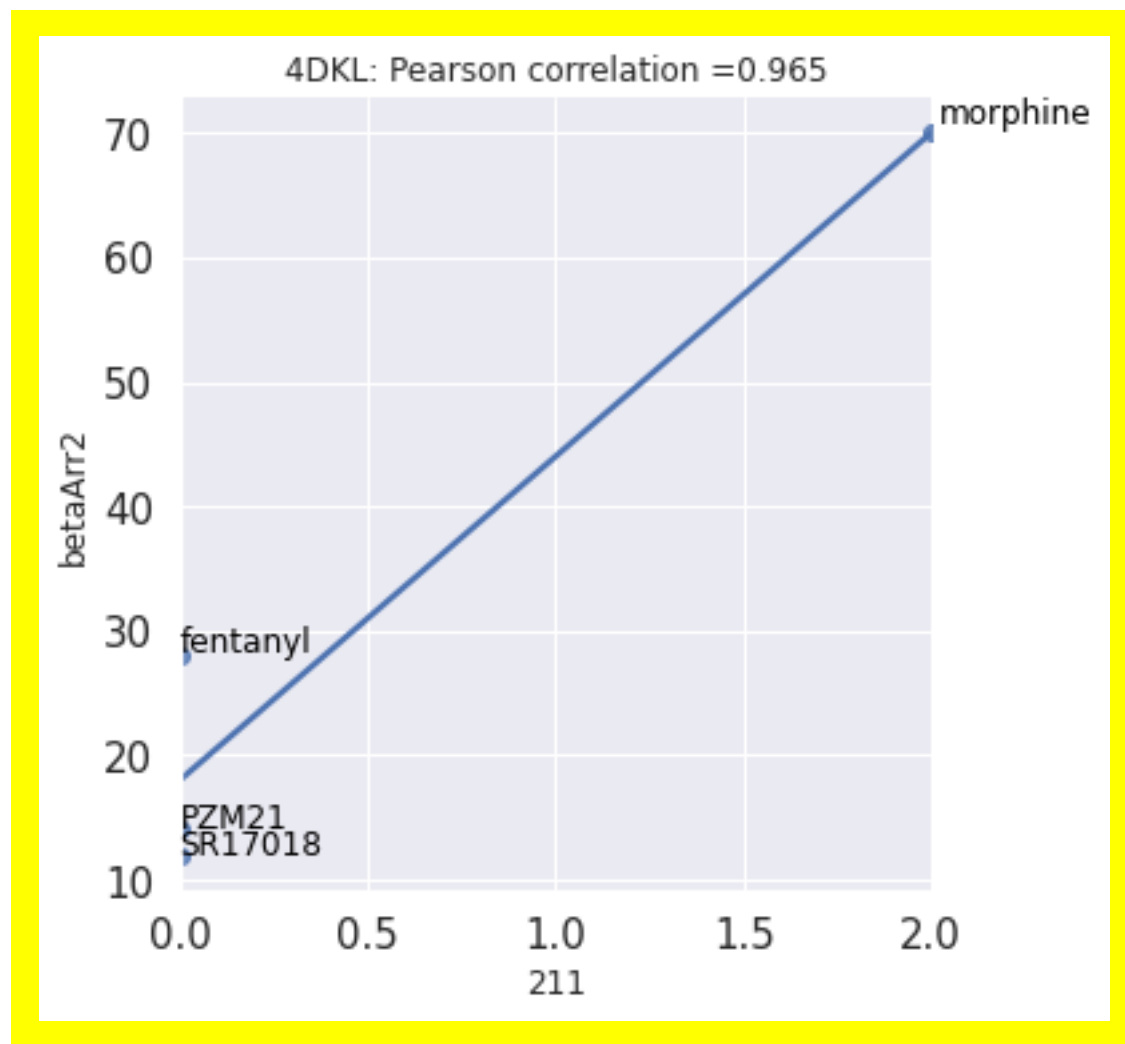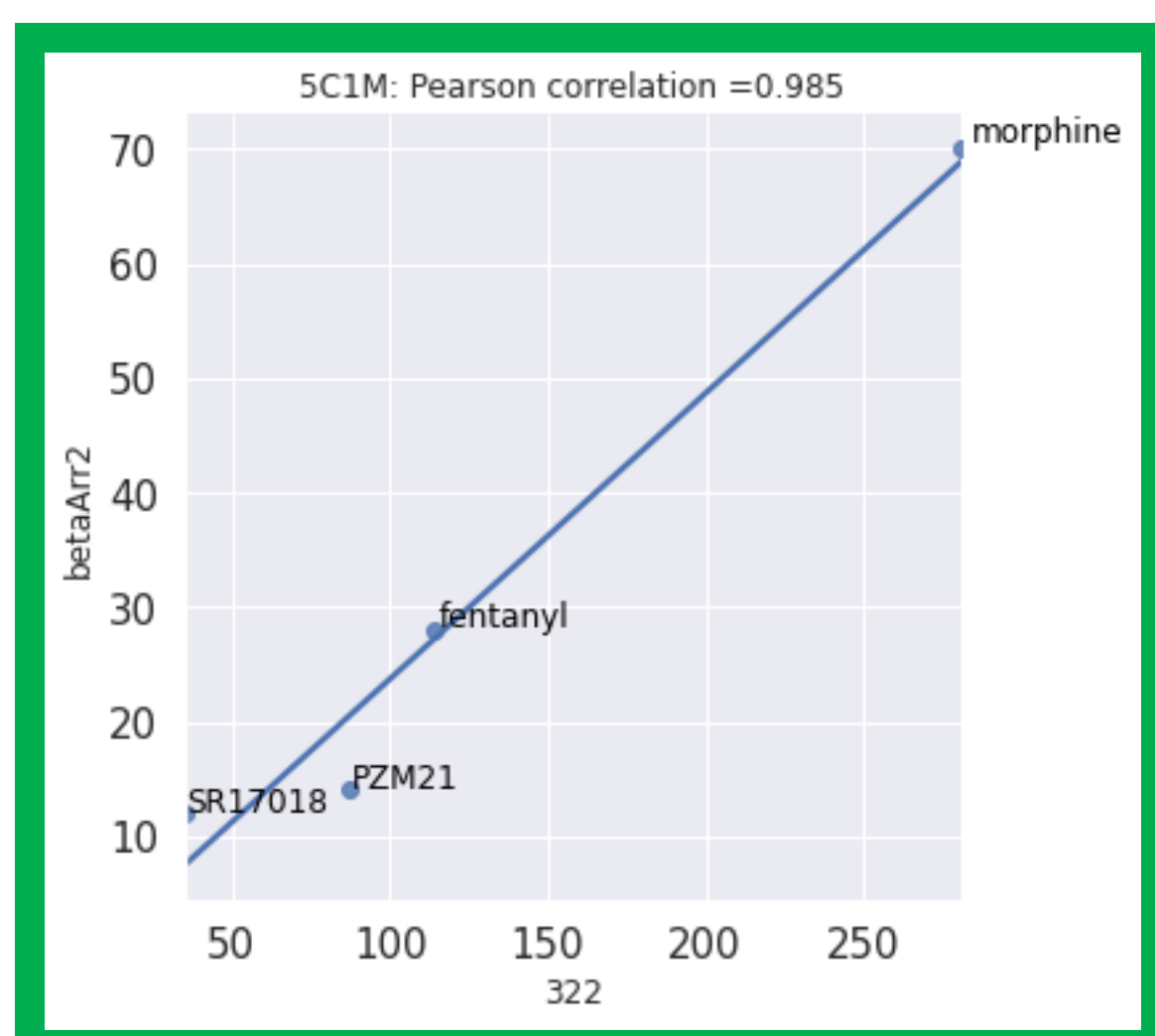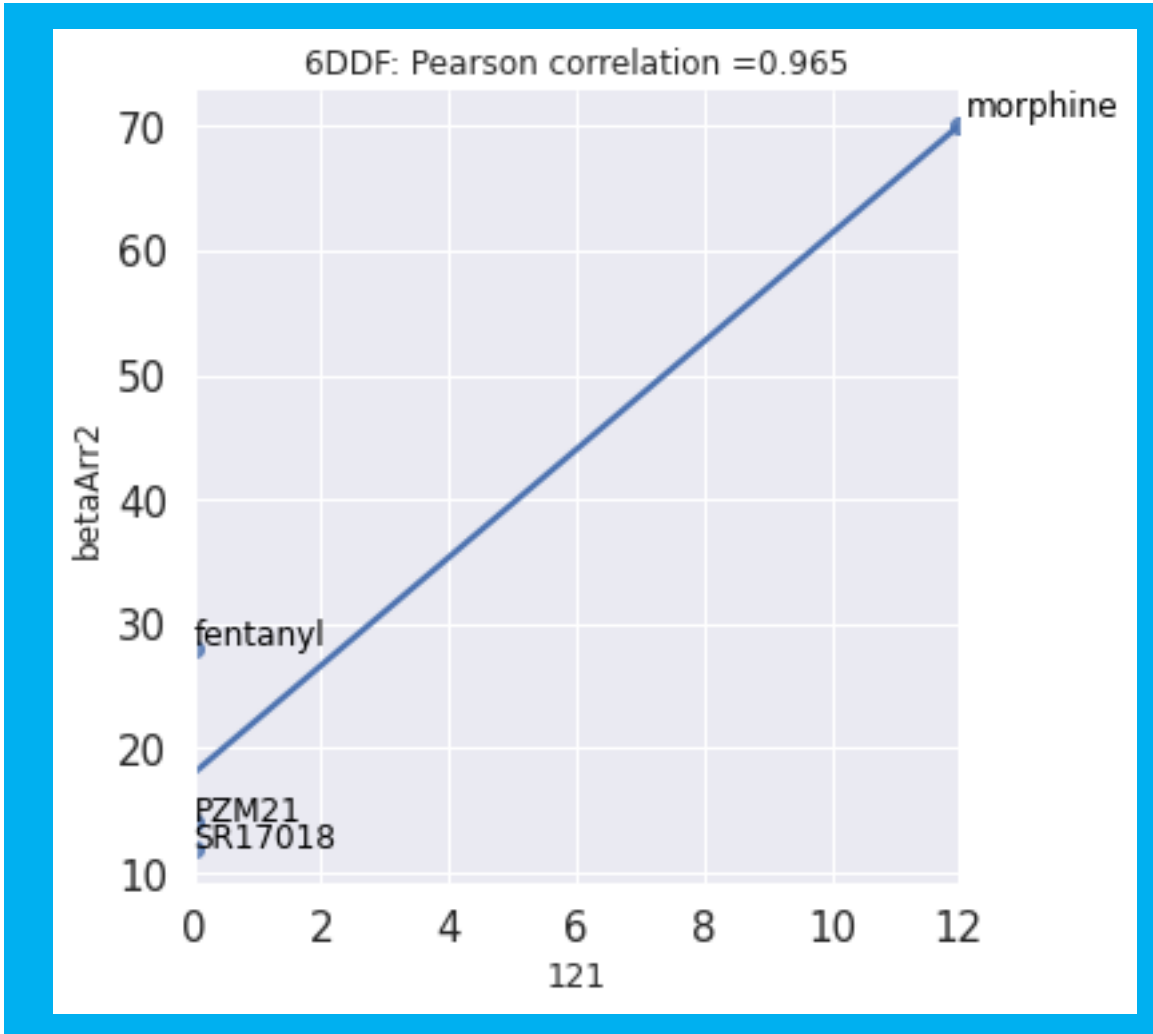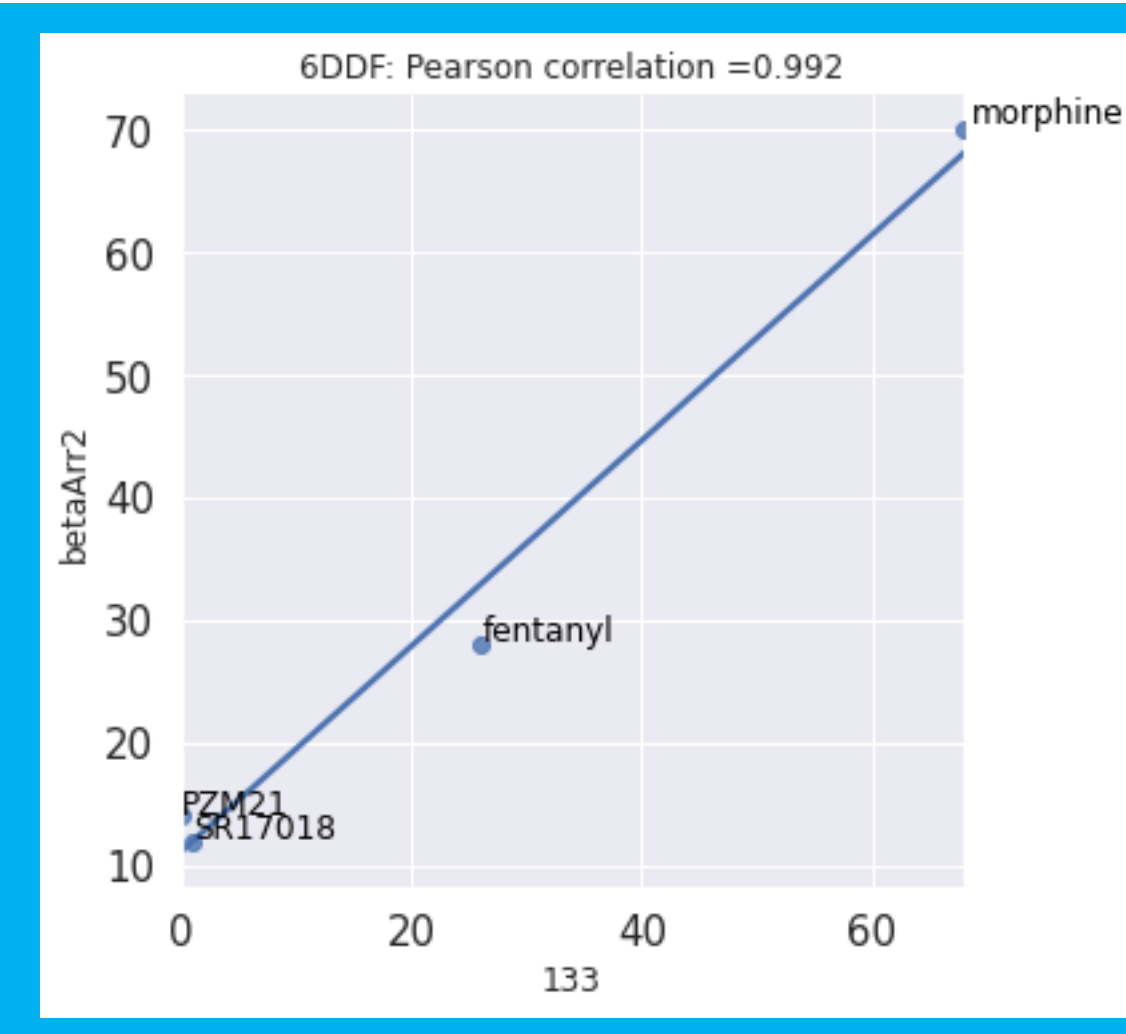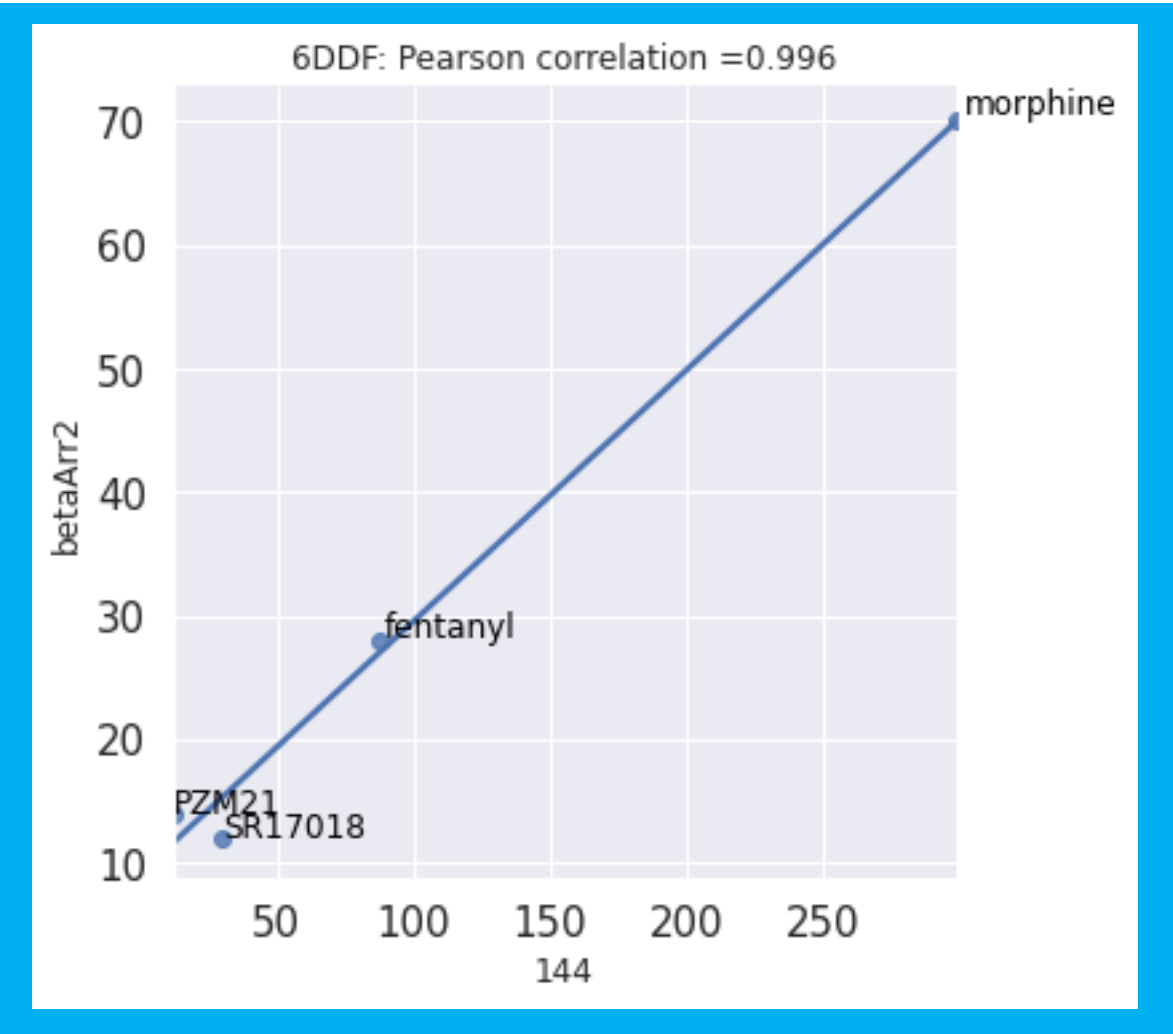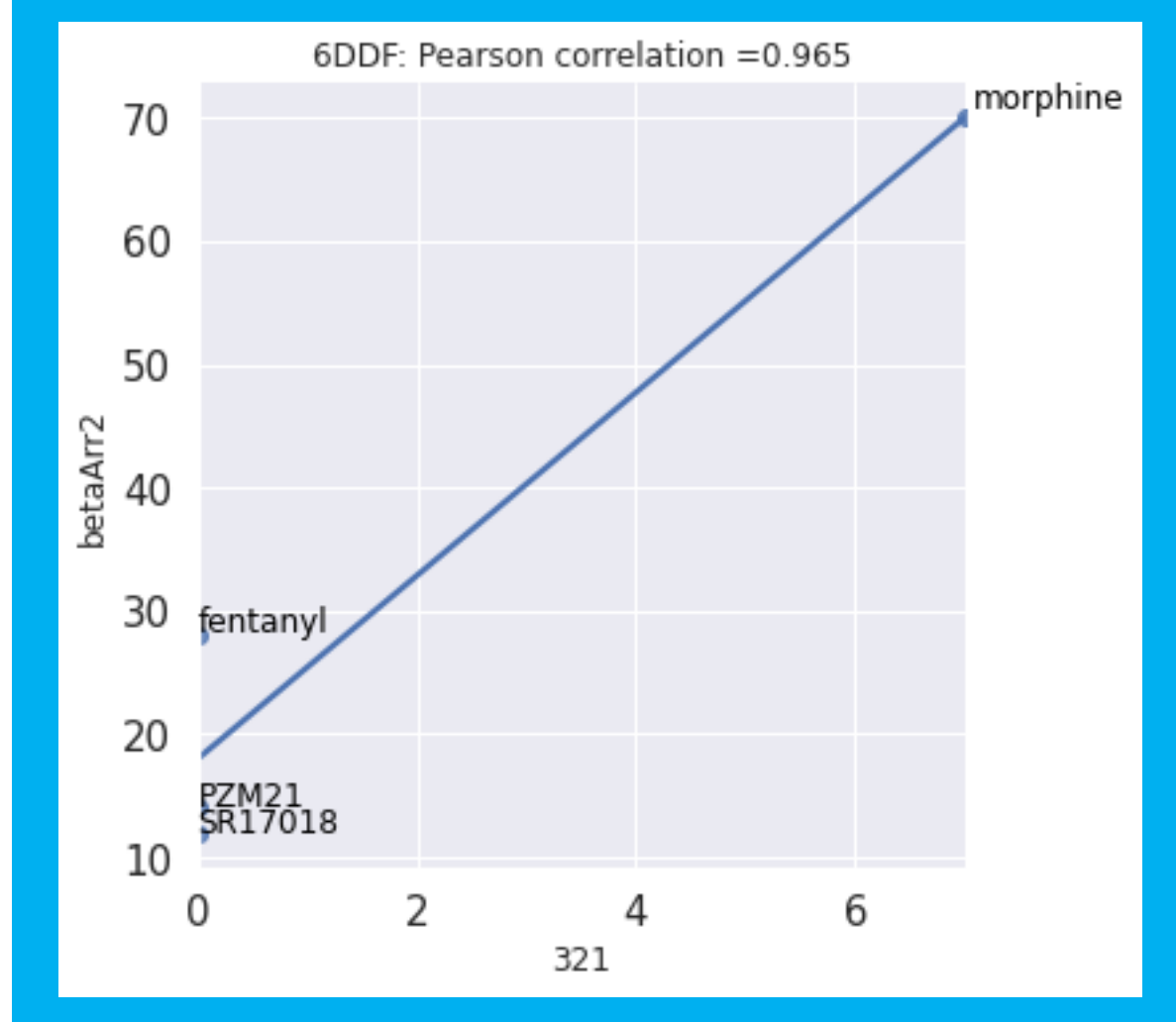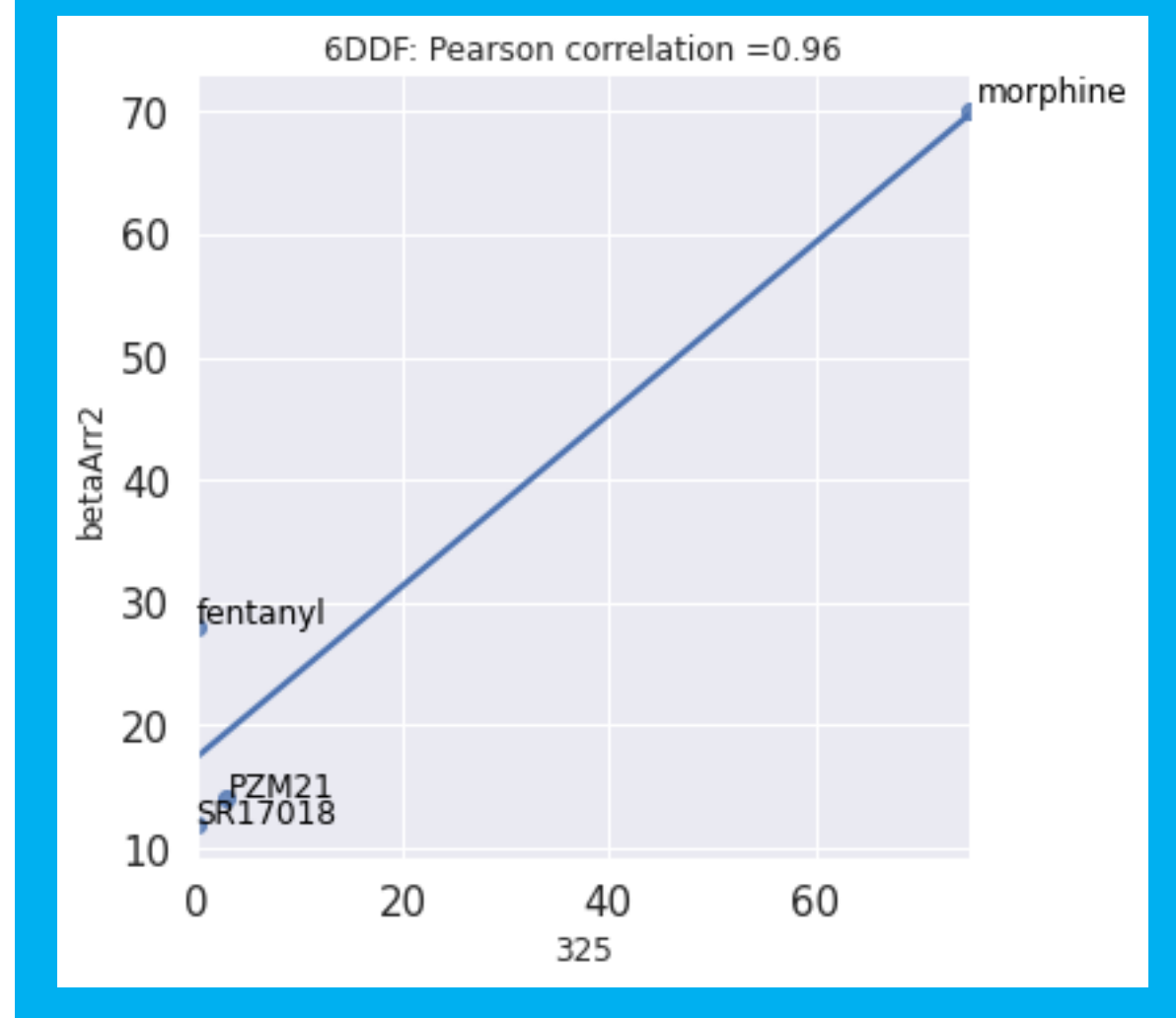

Rab5 trafficking

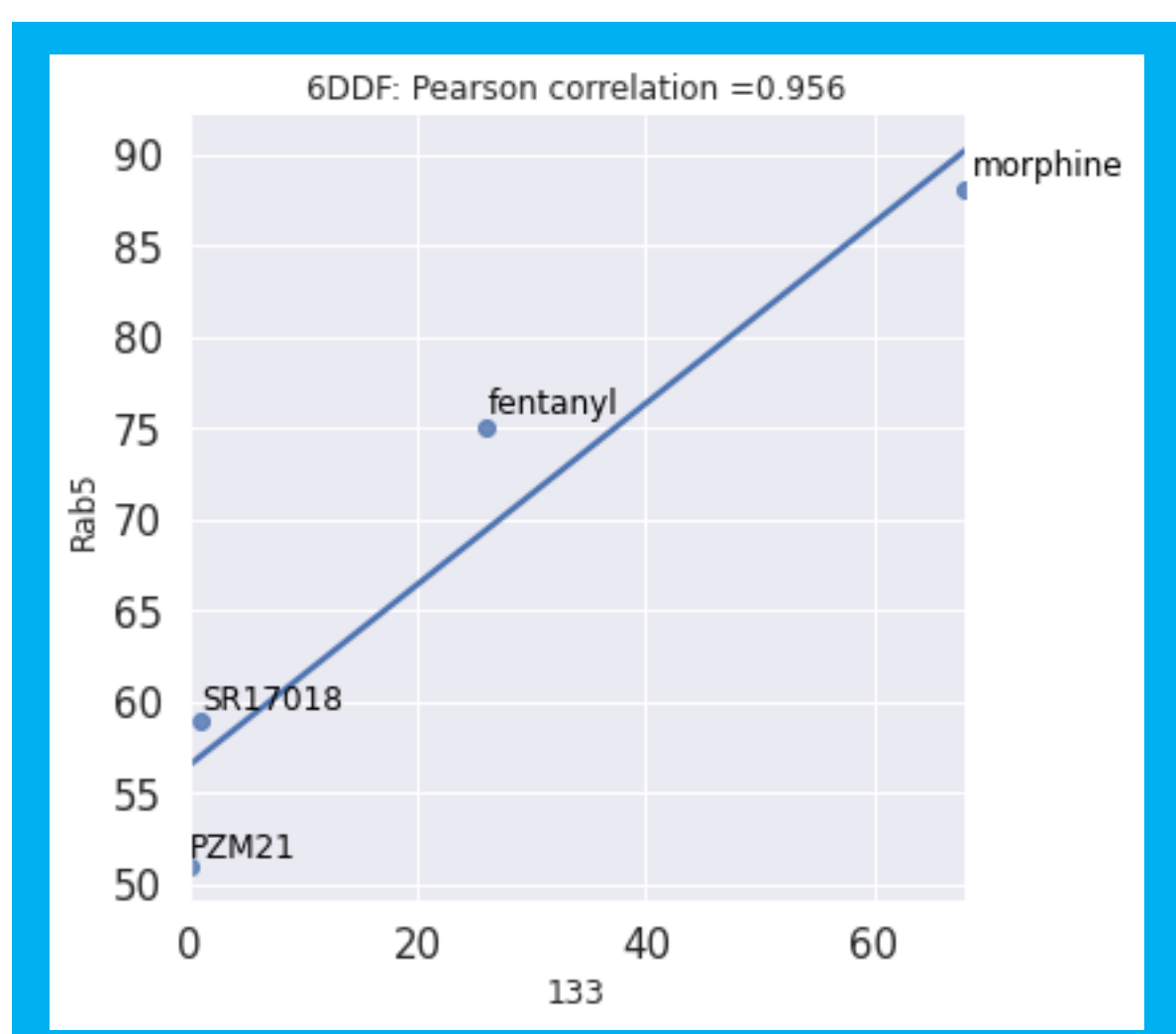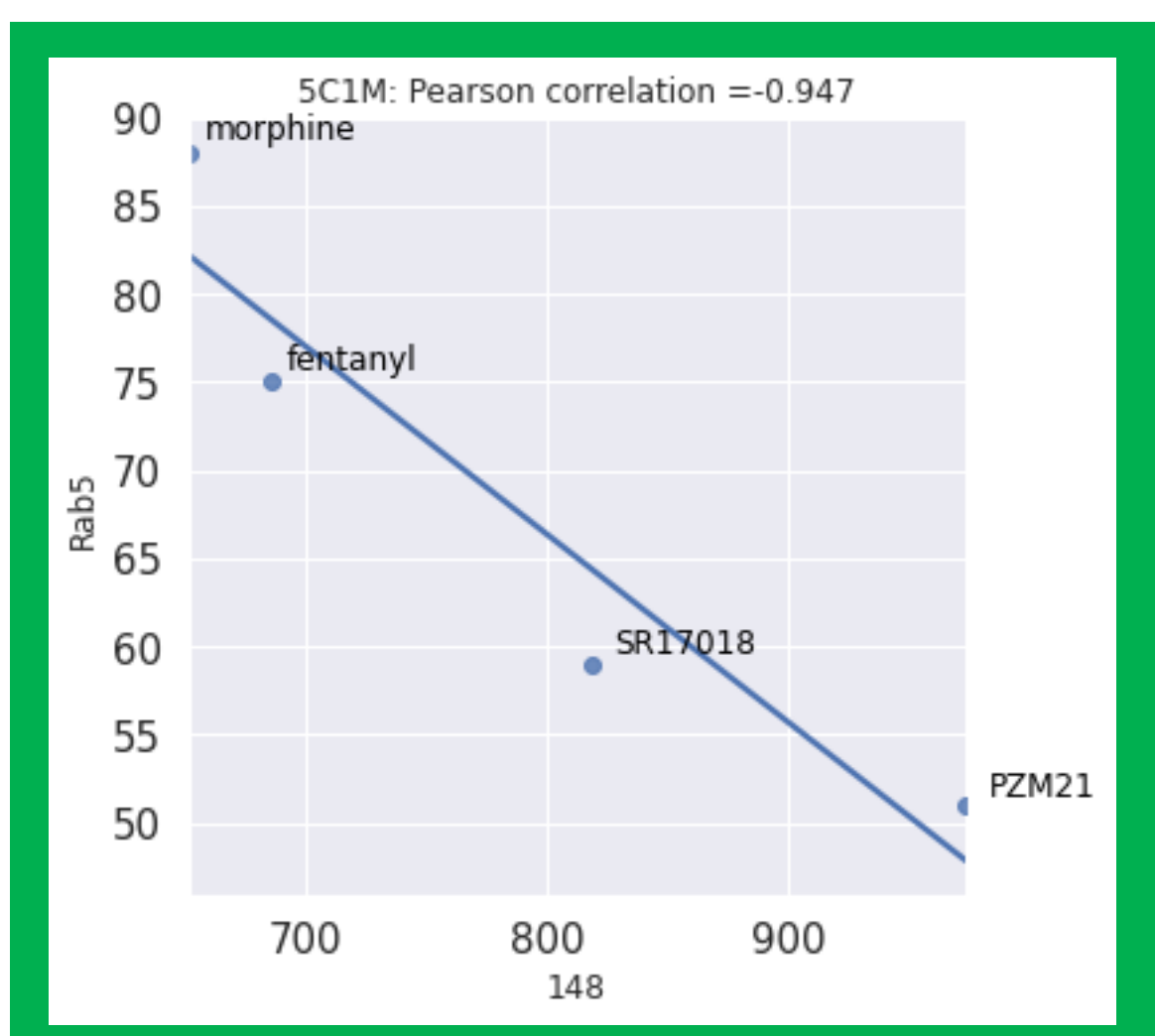

GRK2 recruitment

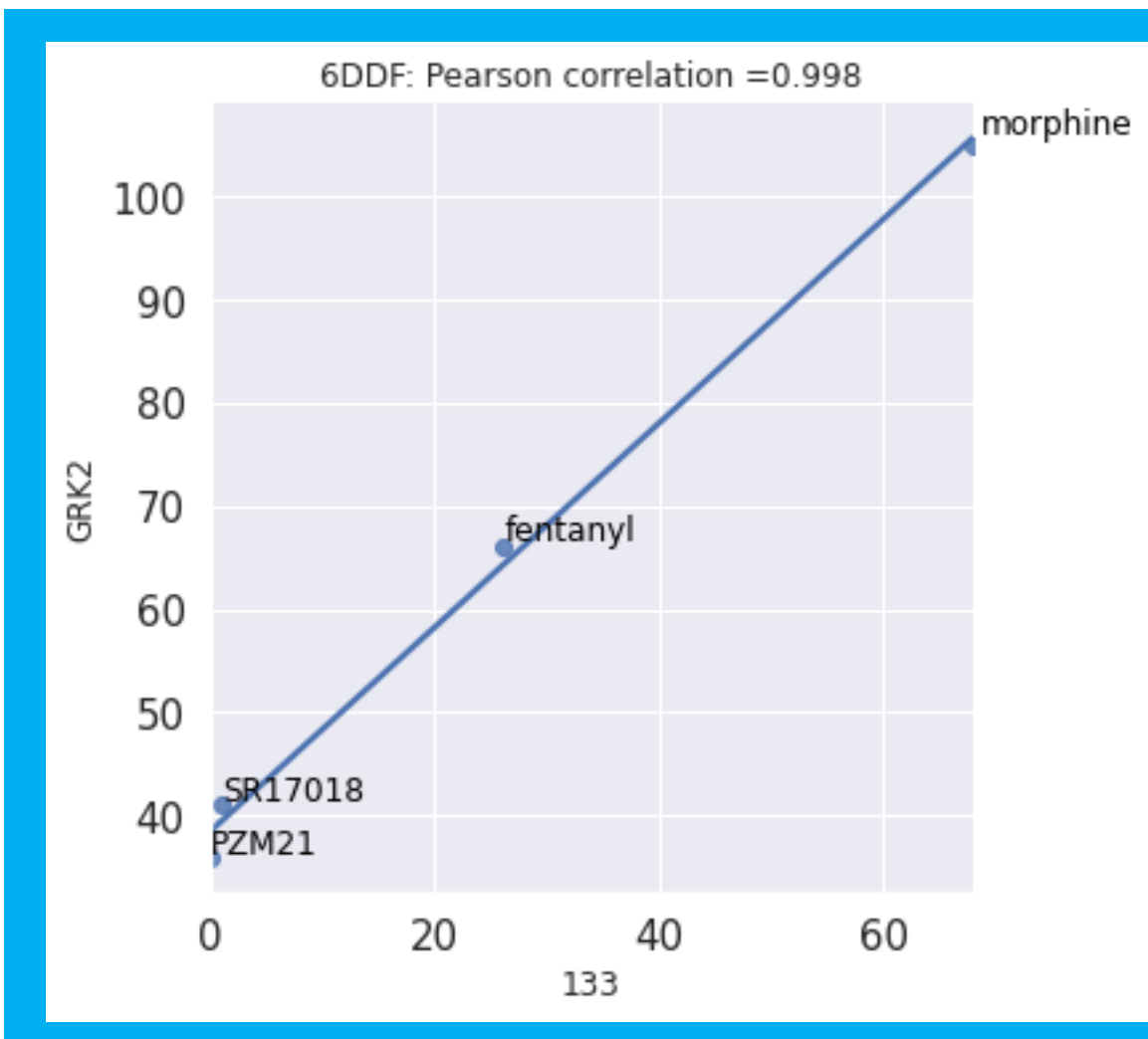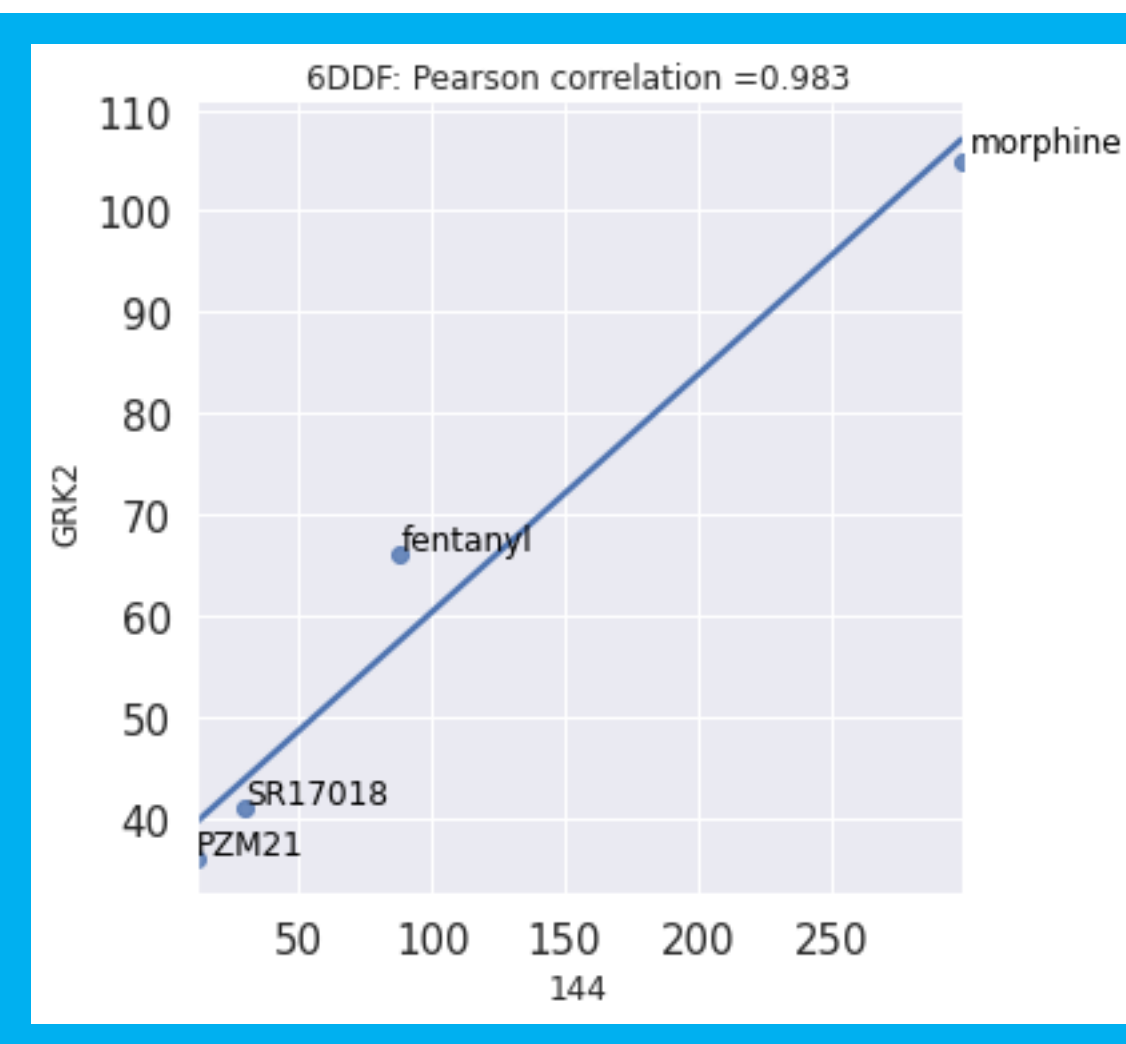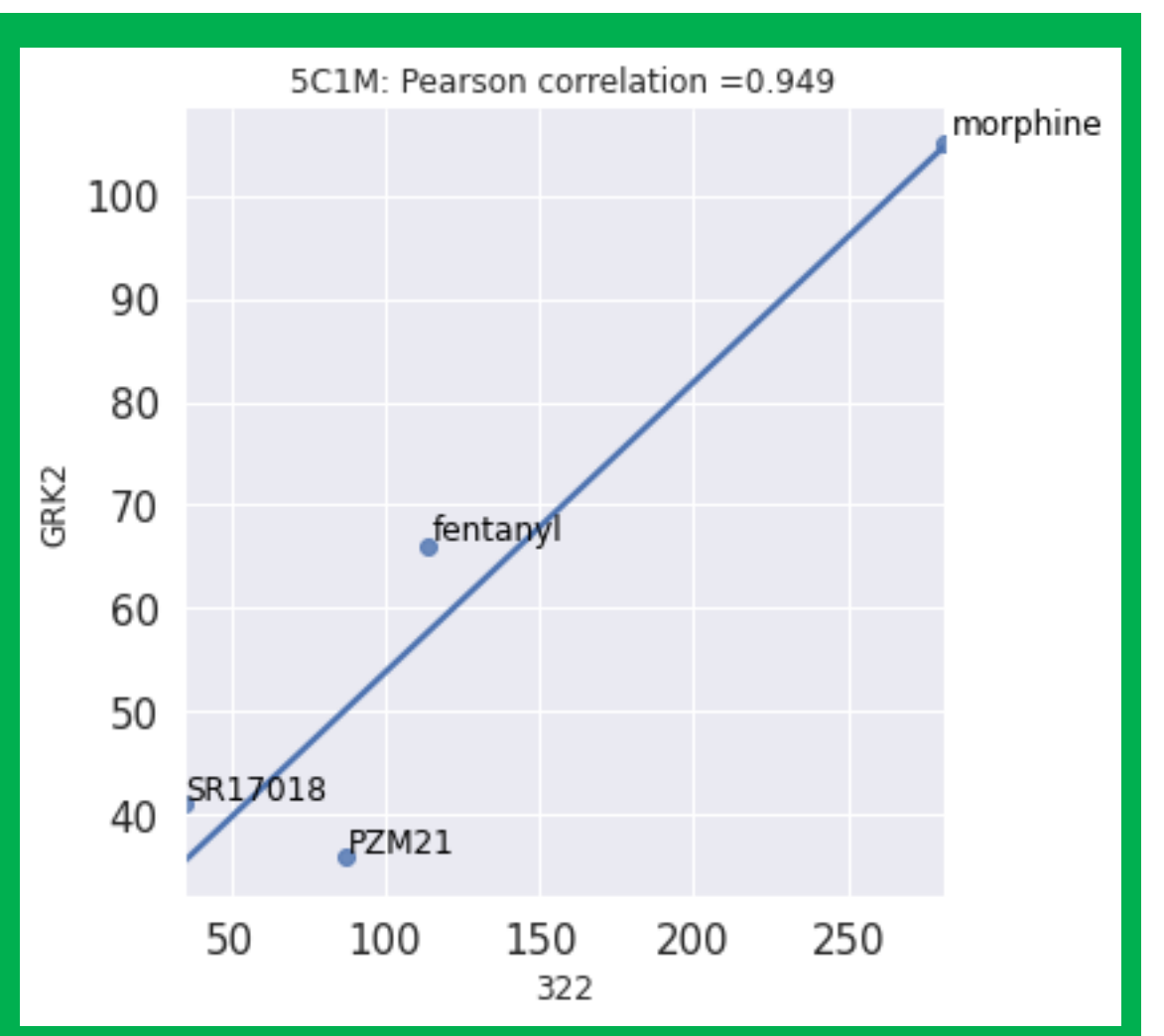

Supplement: Supplementary file 1 [file molecules-25-04636-s001.zip › Figure_S1.pdf]
